# Time-trends for eczema prevalences among children and adults from 1985 to 2015 in China: a systematic review

**DOI:** 10.1186/s12889-022-13650-7

**Published:** 2022-07-05

**Authors:** Wei Liu, Jiao Cai, Chanjuan Sun, Zhijun Zou, Jialing Zhang, Chen Huang

**Affiliations:** 1 Institute for Health and Environment, Chongqing University of Science and Technology, Chongqing, China; 2 School of Environment and Architecture, University of Shanghai for Science and Technology, 516 Jungong Road, Yangpu District, Shanghai, PR China

**Keywords:** Eczema, China, Time-trend, Prevalence, Eczematous population

## Abstract

**Background:**

Several studies have reported that childhood prevalence of eczema has been increasing worldwide. However, none study quantitatively evaluated prevalence trends of eczema among children and adults in the last 30 years in China.

**Methods and Findings:**

Via a systematic review of literature databases in English and Chinese, we summarized all studies reporting eczema prevalences from 1985 to 2015 in China as well as diagramed prevalence and eczematous population trends against year for different age groups. A total of 93 studies and 17 studies (16 for children and one for adults) were selected for qualitative and quantitative synthesis, respectively. Childhood lifetime-ever eczema prevalences ranged from 10.0% to 30.0%. Prevalences among 3-12-year-olds children showed increasing trends in most specific cities, but national lifetime-ever eczema prevalences among 13-14-year-olds children decreased from 10.6% in 2001 to 8.6% in 2009 in mainland China. We estimated that about 1.5 million children aged 13-14-year-olds in 2009 and 15.5 million children aged 3-6-year-olds in 2012 had lifetime-ever eczema in mainland China. Similar studies were too few to ascertain time-trends of eczema prevalence among adults. About 39.4, 20.0, and 11.6 million adults aged 15-86-year-olds in 2010 had contact dermatitis, seborrheic dermatitis, and atopic dermatitis in the mainland China, respectively.

**Conclusions:**

The burden of eczema became heavier in young children, whereas perhaps had been reduced in adolescent in China. More studies for eczema prevalence in adults are warranted.

**Supplementary Information:**

The online version contains supplementary material available at 10.1186/s12889-022-13650-7.

## Introduction

Eczema, also known as dermatitis, is a common skin disease among children and adults [1]. Several studies worldwide have reported childhood and adult prevalences of eczema and their time-trends in the recent years [2–4]. The International Study of Asthma and Allergies in Childhood (ISAAC) reported that in the seven years between Phase One during 1995 and Phase Three during 2002-2003, eczema prevalences in most centers with low prevalence in 1995 had substantially increased, but had mostly leveled or decreased in the 1995 high prevalence countries [2, 3]. The increases in eczema prevalences were more common in the 6-7 than in the 13-14-year-olds age-group [2, 3]. A systematic review of epidemiological studies concluded that childhood prevalence of eczema increased between 1990 and 2010 in Eastern Asia, Western Europe, parts of Northern Europe, and Africa, but no clear trends were identified in other regions; and there were inadequate data for eczema worldwide [4].

Some studies have reported increasing childhood and adult eczema prevalences in China [5–11]. Three large-scale cross-sectional studies from Taichung, Taiwan found that the prevalence of atopic eczema increased from 1.1% in 1987 to 1.9% in 1994, and then to 3.4% in 2002 among 6-15-year-olds children [8]. Two large-scale cross-sectional studies from Taiwan also found that the overall prevalence rate of atopic eczema among 12-15-year-olds adolescents increased from 2.4% during 1995-1996 to 4.0% in 2001; the increase in boys was from 2.7% to 4.2%, and in girls from 2.2% to 3.9% [11].

However, no study has summarized and compared national distributions of eczema prevalences from studies of eczema and no study has evaluated quantitative changes of eczema prevalence trends among children and adults in China. Therefore, in the present review we systematically summarized all studies of eczema prevalences from 1985 to 2015, and quantitatively analyzed trends of eczema prevalences among children and adults in China. We also quantitatively estimated the national eczema prevalence time-trend, and estimated time-trends for absolute numbers of eczematous populations during 1985 to 2015 in the mainland China.

## Methods

A systematic approach was applied to completely collect and select studies with regard to eczema prevalences from 1985 to 2015 in China. First, a literature search was conducted in both English and Chinese databases. Second, we classified all related studies from the literature search into different types, and then the national eczema prevalences for children and adults were estimated according to the selected studies among these studies. Third, we obtained the national absolute populations from China's Statistics Yearbooks for various age groups. Then we estimated the national burdens of childhood and adulthood eczema as equal to national eczema prevalences multiplied by national absolute populations. The detailed approach for literature search and inclusion as well as statistical analyses were as follows.

### Literature search strategy

**Fig 1:**
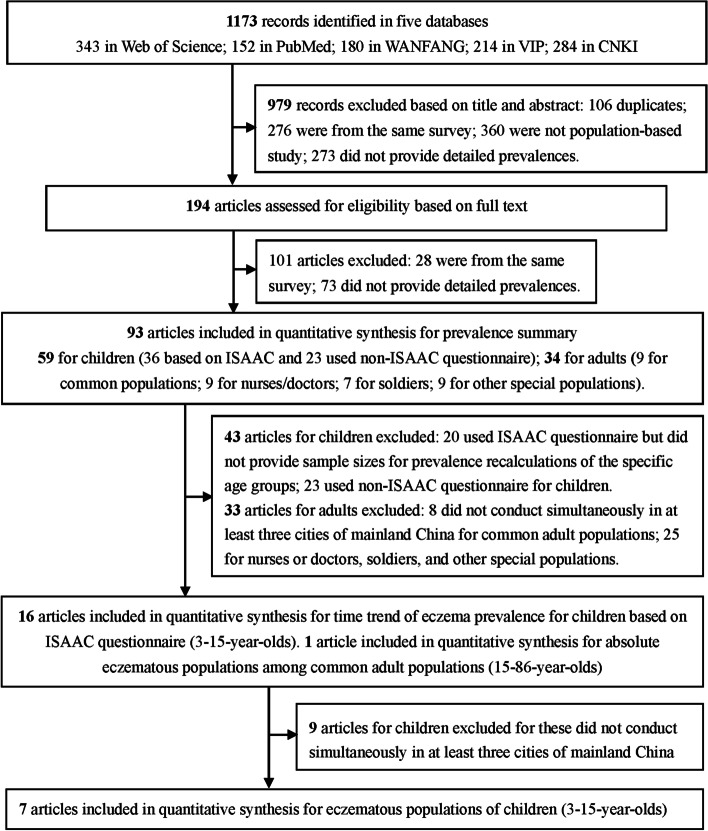
Flow chart of literature search through different phases

The literature search was conducted using PubMed and Web of Science in English, as well as in CKNI (Chinese Knowledge National Infrastructure; in Chinese: Zhong Guo Zhi Wang), VIP (Database for Chinese Technical Periodicals; Wei Pu), and WANFANG DATA (Wang Fang Shu Ji) in Chinese. In PubMed, we used eczema or dermatitis, prevalence or incidence, and China or Hong Kong or Taiwan in title or abstract as search terms (retrieval combination: {[eczema (Title/Abstract) OR dermatitis (Title/Abstract)] AND [prevalence (Title/Abstract) OR incidence (Title/Abstract)] AND [China (Title/Abstract) OR Hong Kong (Title/Abstract) OR Taiwan (Title/Abstract)]}). In Web of Science, we used eczema or dermatitis, prevalence or incidence, and China or Hong Kong or Taiwan in theme subject (TS) as search terms (retrieval combination: TS = (eczema OR dermatitis) AND TS= (prevalence OR incidence) AND TS = (China OR Hong Kong OR Taiwan)). In CKNI, VIP, and WANFANG DATA, we used eczema (in Chinese: Shi Zhen) or dermatitis (Pi Fu Yan) in title, and prevalence (Huan Bin Lü) or incidence (Fa Bin Lü) in abstract as search terms. In CNKI, the retrieval combination was “Title (Pian Ming): eczema (Shi Zhen) or (Huo Han) dermatitis (Pi Fu Yan) + Abstract (Zhai Yao): prevalence (Huan Bin Lü) or (Huo Han) incidence (Fa Bin Lü)”. In VIP, the retrieval combination was “(T = eczema (Shi Zhen) + T = dermatitis (Pi Fu Yan))*(R = prevalence (Huan Bin Lü) + R = incidence (Fa Bin Lü))”. In WANFANG DATA, the retrieval combination was that “(Title (Ti Ming): “eczema (Shi Zhen)” + “dermatitis (Pi Fu Yan)”)* (Abstract (Zhai Yao): “prevalence (Huan Bin Lü)” + “incidence (Fa Bin Lü)”)”. Figure 1 is a flowchart of papers through the selected phases. We also retrieved references of the selected studies. Finally, 194 full-text articles from January 1985 to December 2015 were identified after removing duplicates. Herein 146 articles were in English and 48 articles in Chinese.

### Inclusion criteria

In the quantitative synthesis, we summarized all questionnaire-based cross-sectional and cohort studies as well as clinical studies that reported childhood or adult eczema prevalence. The studies were classified into six types: ISAAC questionnaire-based studies of children, non-ISAAC questionnaire-based studies of children, studies of overall adult populations, studies of specific adult populations, namely, nurses and/or doctors, soldiers, and other special populations. Then, two reviewers independently quality-checked the studies, and selected those questionnaire-based cross-sectional studies of children for quantitative synthesis when they met the following criteria: 1) conducted on the general population in the studied city; and 2) based on the ISAAC questionnaire [12] or any questionnaire that used the question “Have your children (or you) had eczema (an itchy skin rash coming and going for at least 6 months) during lifetime-ever (or in the last 12 months)”. In addition to these two criteria, in the quality assessment of the selected studies, we also checked sample size and response rate to consider their quality. If a study matched the above two criteria, obtained enough samples that were higher than the estimated sample size, and had high (>70%) response rate in the survey, we considered that this study had high quality. For time-trends, we selected those cities where similar studies were conducted in at least two different years of children in the same age groups. We also selected those studies which provided sufficiently detailed sample numbers to calculate the childhood eczema prevalence in different age groups. To elucidate national time-trends and estimate national absolute eczematous populations, we selected only those studies conducted simultaneously in at least three cities of mainland China.

### Statistical analyses

Microsoft Excel 2013 (Microsoft Ltd. Seattle, Washington, USA) was used to calculate city-level and national-level prevalence time-trends and absolute eczematous proportions in children and adults. A pooled analysis was conducted to obtain nation-level and city-level eczema prevalences in various years, and these prevalences were estimated to be equal to that of the cumulative number of eczematous children/adults divided by the cumulative total sample sizes in different cities for the given year. The absolute eczematous populations in different age groups were estimated as equal to national eczema prevalences multiplied by the national absolute populations, obtained from China's Statistics Yearbooks during 1996-2014 [13] for various age groups. China’s Statistics Yearbooks provided only the populations of mainland China, and not of Hong Kong or Taiwan. China's Statistics Yearbook also did not present detailed age-group populations for children and adults in the total national populations of mainland China for a specific year. For all years, the yearbooks did provide populations in the age groups 0-14 years, 15-64 years, and ≥65 years for 1996 to 2000, and for 2001 to 2014 provided data for 0-5, 6-9, 10-14,…, 85-89, 90-95, ≥95 years. Therefore, we assumed that the proportions of populations for each specific age were the same within various age groups, and calculated these proportions as the total proportions divided by the total number of years of the studied age groups [8, 14]. The specific population of children and adults at each specific age was calculated as the proportion of population in each specific age group multiplied by the national population. Then the national absolute populations for various age groups were summed as these specific populations for each specific age group.

## Results

### Basic Information

A total of 93 articles was finally selected for quantitative synthesis [5–11, 15–100]. Of these, 36 studies were cross-sectional studies, based on cluster sampling in which ISAAC questionnaires were used [5, 7–11, 15–44]; and 23 studies of children did not use the ISAAC questionnaire [6, 45–66]. There were 9 studies of adult populations [67–75]; 9 studies of nurses or doctors [76–84]; 7 studies of soldiers [85–91]; and 9 studies of other special populations [92–100]. More information for these studies is summarized in supplemental S1-S7 Tables. A total of 17 studies was selected to quantitatively analyze time trends of eczema prevalence and absolute eczematous populations in different age groups. Of these, sixteen studies were of children [5, 7–9, 11, 15–18, 24–27, 29, 35, 44], and one of adults [74] (Table 1). Only one study provided eczema prevalence for each specific age of 0-14-year-olds children [7]. Seven studies [5, 7, 16, 24–26, 35] were selected to quantitatively estimate the absolute eczematous populations among children and one for adults [74].

**Table 1:** Basic information for studies included in quantitative analyses of absolute eczematous populations and prevalence time trends

Year	Age (year-old)	Location and Inspection method	City: Sample size (response rate)	Prevalence, *n* (%)	[Reference]
1987	7-15	School; Student/parent-reported	Taichung, Taiwan: 37801 (78.0)	Ever: 420 (1.11); Current: 333 (0.88)	[8]
1994	7-15	School; Student/parent-reported	Taichung, Taiwan: 75960 (83.0)	Ever: 1428 (1.9); Current:1155 (1.5)	[8]
1994	6-7	Elementary school; Parent-reported	Taipei, Taiwan: 4806 (NA)	Ever: 1149 (23.9); Past year: 197 (4.1)	[9]
1995	13-14	Secondary school; Parent-reported	Hong Kong: 4667 (97.0)	Ever: 707 (15.0); Past year: 210 (4.5)	[15]
1995	13-14	School; Student-reported	Beijing: 4167 (99.0) Guangzhou: 3855 (99.6); Urumqi: 3207 (98.0); Shanghai: 3483 (99.0); Chongqing: 4296 (99.0)	Beijing: Ever: 429 (10.3); Past year: 958 (2.3). Guangzhou: Ever: 705 (18.3); Past year: 50 (1.3). Urumqi: Ever: 196 (6.1); Past year: 67 (2.1). Shanghai: Ever: 240 (6.9); Past year: 42 (1.2). Chongqing: Ever: 434 (10.1); Past year: 86 (2.0)	[16]
1995	6-7	Primary school; Parent-reported	Hong Kong: 3618 (97.0)	Ever: 1017 (28.1); Past year: 152 (4.2)	[17]
1996	6-7	Primary school; Parent-reported	Beijing: 4080 (99.2); Urumqi: 3588 (98.6)	Beijing: Ever: 155 (3.8); Past year: 114 (2.8). Urumqi: Ever: 126 (3.5); Past year: 72 (2.0)	[18]
1996	12-15	Middle school; Parent-reported	Taiwan^c^: 42919 (86.9)	Past year: 10472 (2.4)	[11]
2001	12-15	Middle school; Parent-reported	Taiwan^c^: 10215 (87.0)	Past year: 412 (4.0)	[11]
2001	13-14	School; Student-reported	Beijing: 3531 (99.0)	Ever: 434 (12.3); Past year: 64 (1.8)	[24]
2001	13-14	School; Student-reported	Guangzhou: 3675 (96.0)	Ever: 620 (17.6)	[25]
2001	13-14	Junior high-school; Student-reported	Lhasa, Tibet: 3190 (100.0)	Ever: 45 (1.4); Past year: 12 (0.4)	[26]
2002	13-14	Secondary school; Parent-reported	Hong Kong: 3321 (99.0)	Ever: 421 (13.0); Past year: 120 (3.6)	[29]
2002	6-7	Elementary school; Parent-reported	Taipei, Taiwan: 4832 (NA)	Ever: 1271 (26.3); Past year: 411 (8.5)	[9]
2002	6-7	Primary school; Parent-reported	Hong Kong: 4448 (95.0)	Ever: 1366 (30.7); Past year: 187 (4.2)	[27]
2002	7-15	School; Student/parent-reported	Taichung, Taiwan: 11580 (81.0)	Ever: 388 (3.4); Current: 322 (2.8)	[8]
2005^a^	6-13	Elementary school; Parent-reported	Harbin: 2900 (NA); Shanghai: 4395 (NA); Guangzhou: 3094 (NA); Xi'an: 1653 (NA); Wuhan: 2061 (NA); Chengdu: 2848 (NA); Hohhot: 2025 (NA); Urumqi: 2033 (NA)	Harbin: Past year: 136 (4.7). Shanghai: Past year: 286 (6.5). Guangzhou: Past year: 167 (5.4). Xi'an: Past year: 73 (4.4). Wuhan: Past year: 126 (6.1). Chengdu: Past year: 122 (4.3). Hohhot: Past year: 130 (6.4). Urumqi: Past year: 120 (5.9).	[35]
2007	6-7	Elementary school; Parent-reported	Taipei, Taiwan: 24999 (94.6)	Ever: 7450 (29.8); Past year: 2675 (10.7)	[9]
2009	0-14	School/kindergarten; Parent-reported	Beijing: 10372 (98.6); Chongqing: 9846 (97.21); Guangzhou: 4072 (90.9)	Beijing: Ever: 2141 (20.6). Chongqing: Ever: 1085 (10.0). Guangzhou: Ever: 294 (7.2).	[7]
2012	3-6	School/kindergarten; Parent-reported	Harbin: 2506 (64.1); Urumqi: 4618 (81.7); Beijing: 5876 (65.0); Shanghai: 15266 (85.3); Nanjing: 4014 (65.7); Xi'an: 2020 (83.5); Taiyuan: 3700 (82.2); Wuhan: 2193 (91.4); Changsha: 2727 (59.0); Chongqing: 5299 (74.5)	Harbin: Ever: 829 (33.1); Past year: 306 (12.2). Urumqi: Ever: 707 (15.3); Past year: 614 (13.3). Beijing: Ever: 2039 (34.7); Past year: 928 (15.8). Shanghai: Ever: 3572 (23.4); Past year: 2122 (13.9). Nanjing: Ever: 1140 (28.4); Past year: 429 (10.7). Xi'an: Ever: 586 (29.0); Past year: 166 (8.2). Taiyuan: Ever: 503 (13.6); Past year: 178 (4.8). Wuhan: Ever: 570 (26.0); Past year: 184 (8.4). Changsha: Ever: 815 (29.9); Past year: 265 (9.7). Chongqing: Ever: 1611 (30.4); Past year: 684 (12.9).	[5]
2015	7-12	School; Student/parent-reported	Guangzhou: 5542 (94.3)	Ever: 1890 (34.1)	[44]
2010 ^b^	15-86	home-interview, participant-reported and examined by dermatologists [101]	Beijing: 1443 (NA); Shanghai: 6036 (NA); Guangzhou: 1675 (NA)	Beijing: Current dermatitis: contact: 47 (3.3); seborrheic: 29 (2.0); atopic: 22 (1.5). Shanghai: Current dermatitis: contact: 192 (3.2); seborrheic: 75 (1.2); atopic: 38 (0.6). Guangzhou: Current dermatitis: contact: 85 (5.1); seborrheic: 60 (3.6); atopic: 35 (2.1).	[74]

^a^ The response rate for the whole study was 92.5% and The response rate for each city was not available (NA)

^b^ The response rate was not available (NA)

### Quality assessment of the selected studies

With respect to the 16 studies which was selected to analyze time trends and absolute eczematous populations for children (Table 1), all studies were conducted in school; and except the studies in 1995 [16] and in 2001 [24, 25] for 13-14-year-olds children, all questionnaire data were reported by the child’s parents. All studies had large sample sizes (mostly in 2000~5000 children) and most of these studies had high response rates (mostly in >80%). Two studies [9, 35] did not provide available response rates. With respect to the study which was selected to analyze absolute eczematous populations for common adult population (Table 1), the interview was conducted in the participant’s home and the participant-reported eczema (dermatitis) were examined by dermatologists. The study had large sample size in Shanghai (*n*=6036) and had moderate sample sizes in Beijing (*n*=1443) and Guangzhou (*n*=1675). The response rate for this study was not available.

### Childhood eczema

**Fig 2:**
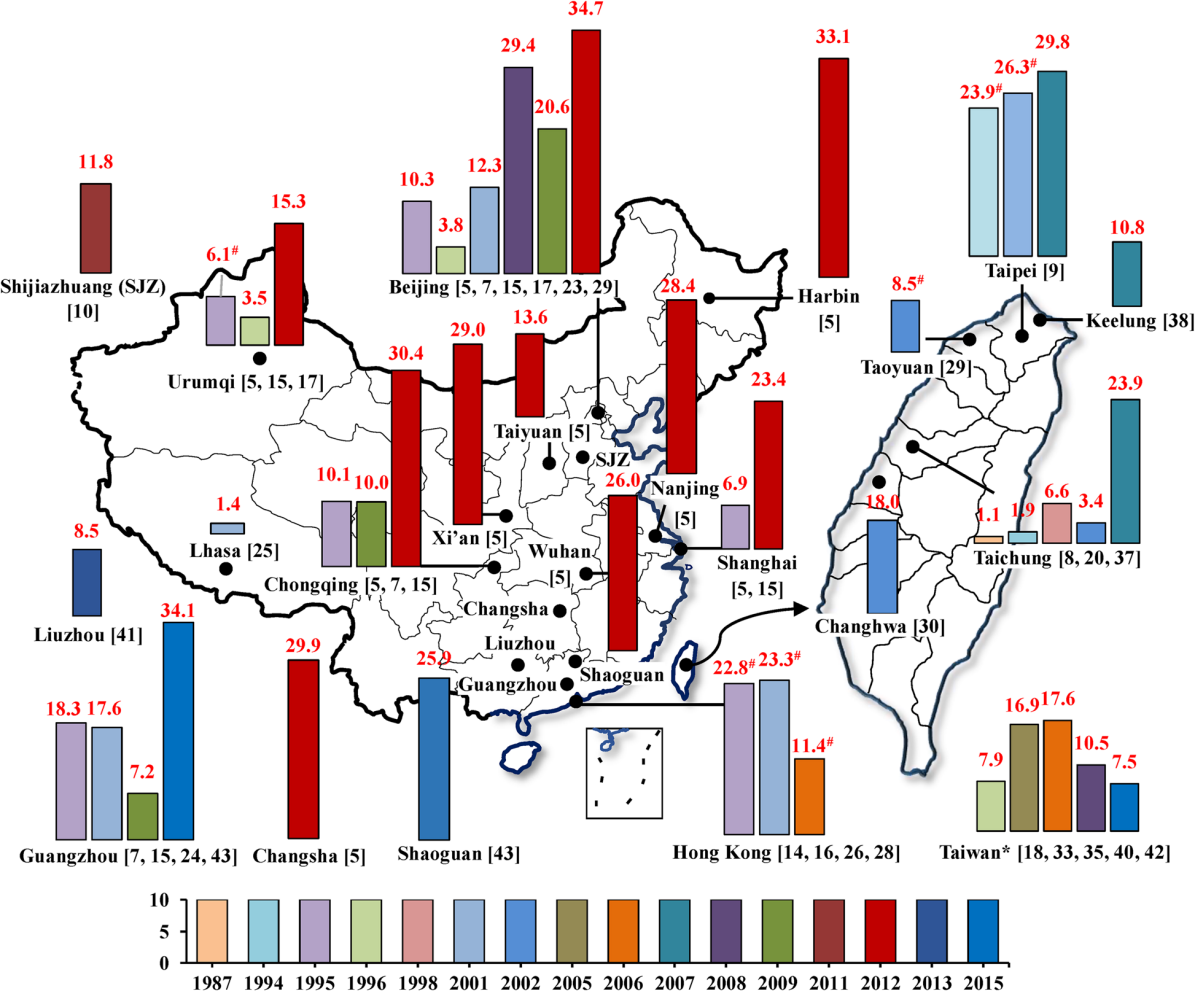
Summary of eczema prevalence during lifetime-ever for children from studies which using the ISAAC questionnaire in China, 1985-2015. Herein “*” indicates that the exact city where the study was conducted was not provided in the literature; “^#^” indicates that the presented prevalence was averaged among prevalences for various age groups from different studies in the same year. Number in the bracket is the reference serial number. Supplemental S1 Table shows more information for these studies. The bar charts were conducted by Microsoft Excel 2013 and Word 2013 (https://www.microsoft.com/en-us/). The based maps of mainland China and Taiwan were generated by ESRI ArcGIS 10.0 version for desktop (http://www.esri.com/software/arcgis/arcgis-for-desktop)

Figure 2 and the supplemental S1 Table summarize prevalences of lifetime-ever eczema for children in different age groups from all ISAAC questionnaire-based cross-sectional studies. A total of 22 cities or regions (15 cities in mainland of China, 5 cities in Taiwan, Hong Kong, and Taiwan region) had available data. Overall, the children were 0-18-year-olds and were mainly 3-14-year-olds. Prevalences mostly ranged from 10.0% to 30.0% but varied from 1.1% in 7-15-year-olds children in 1987 in Taichung, Taiwan [8] to 34.7% in 3-6-year-olds children in 2012 in Beijing [30]. In general, childhood eczema prevalences had an increasing trend in Beijing, Urumqi, Chongqing, Shanghai, Guangzhou, Taipei, and Taichung. Decreasing trends were found for Hong Kong as well as for those studies from “Taiwan” where the studies were conducted without providing the specific studied cities of Taiwan.

**Fig 3:**
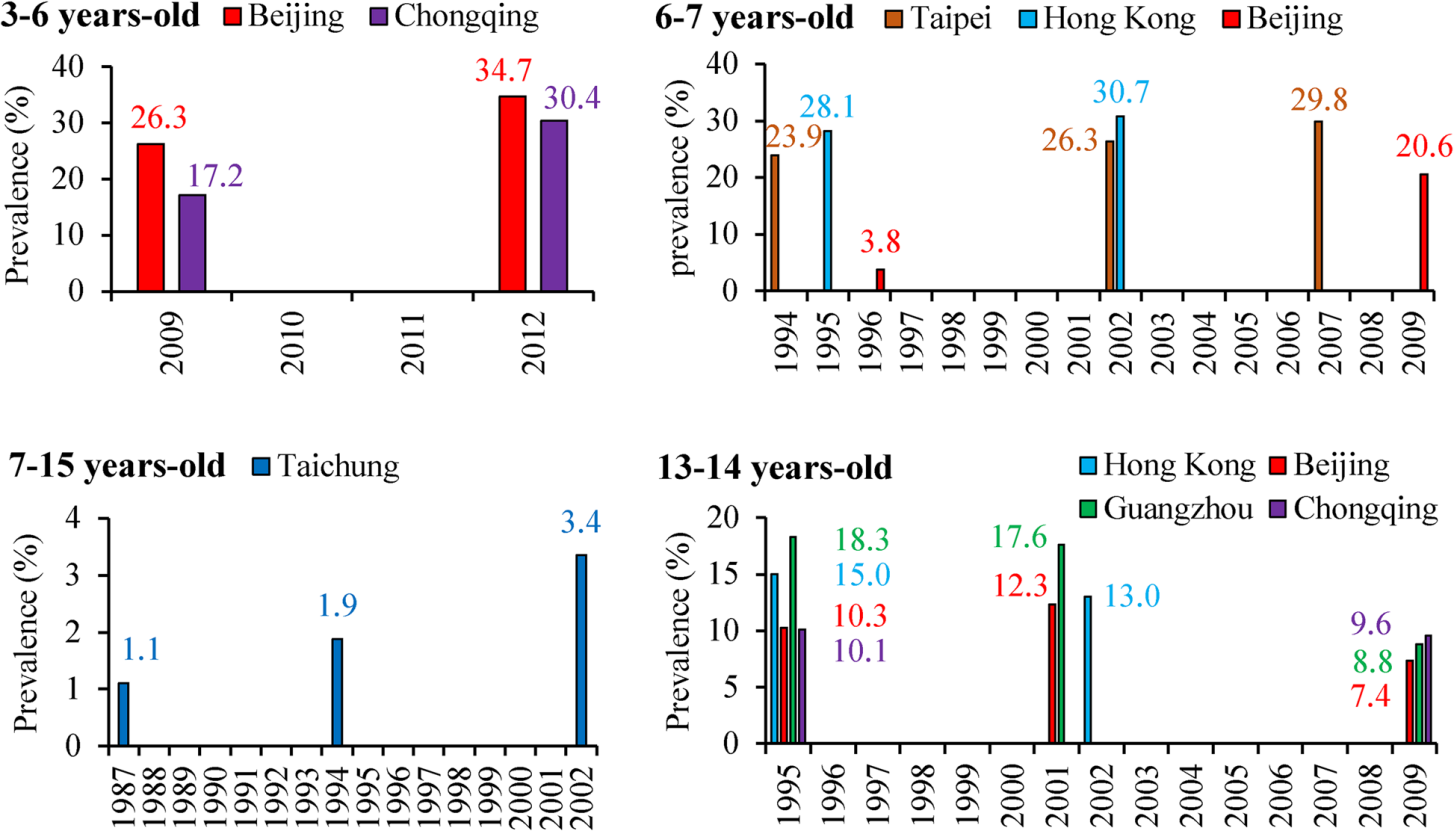
Time-trend of lifetime-ever eczema prevalences among children in specific age groups and cities. The number colors correspond to the bar colors for different cities

Among those cities where data for lifetime-ever eczema were available for children in the sample age groups in more than two years (Fig. 3), eczema prevalences were substantially increased from 1985 to 2015 among children aged 3-6, 6-7, 7-12, and 7-15-year-olds, whereas the eczema prevalences among 13-14-year-olds children consistently decreased from 1995 to 2009. Most of the eczema prevalences in younger age groups (3-7-year-olds) were higher than for older children (13-14-year-olds).

**Fig 4:**
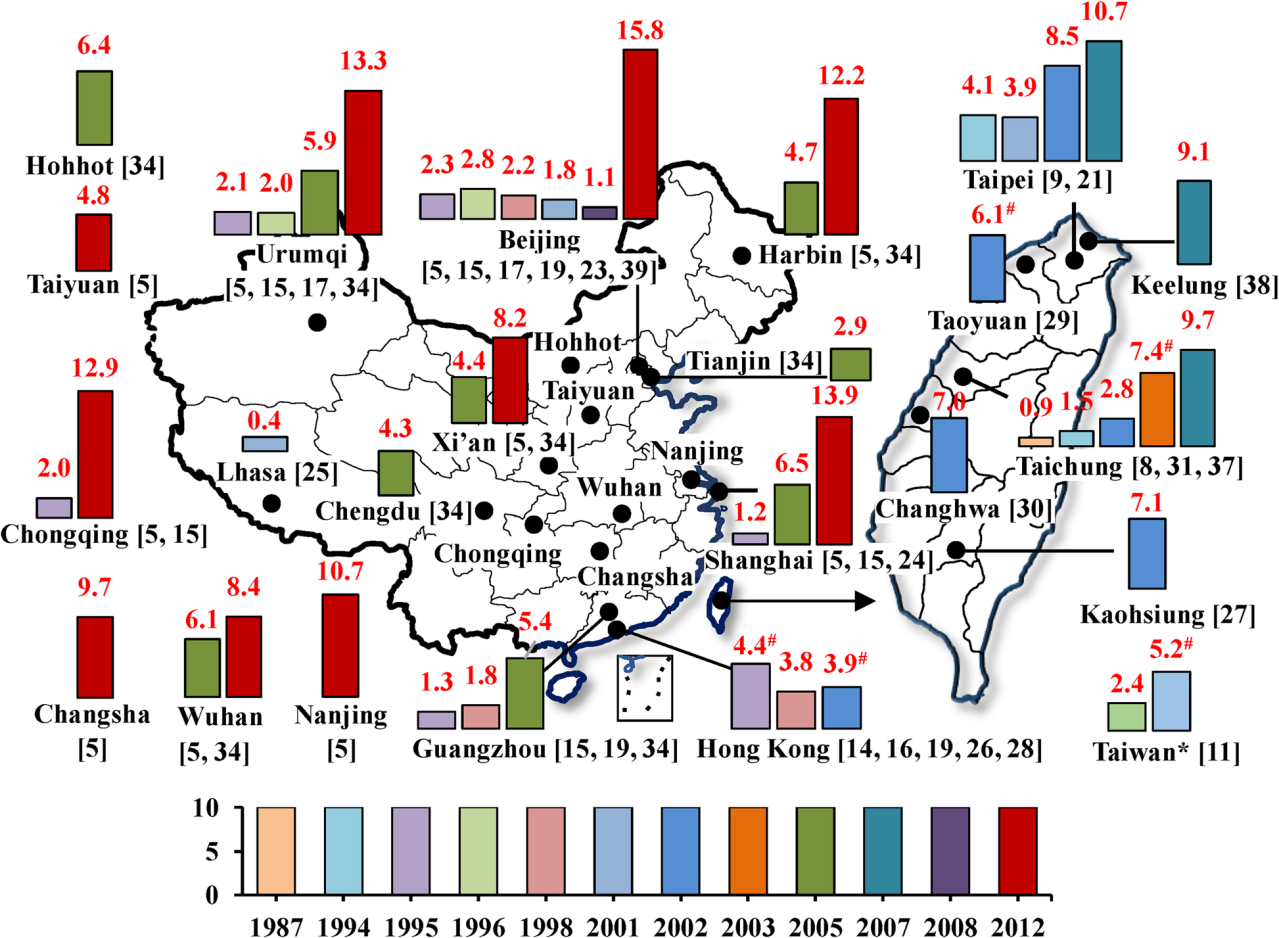
Summary of current (in the past 12 months prior to survey) eczema prevalence among children from studies based on the ISAAC questionnaire in China, 1985-2015. Herein “*” indicates that the exact city was not reported in the literature; “^#^” indicates that the presented prevalence was the average of prevalences in various age groups from different studies in the same year. The number in the bracket is the reference number. Supplemental S1 Table provides more information on these studies. The bar charts were conducted by Microsoft Excel 2013 and Word 2013 (https://www.microsoft.com/en-us/). The based maps of mainland China and Taiwan were generated by ESRI ArcGIS 10.0 version for desktop (http://www.esri.com/software/arcgis/arcgis-for-desktop)

**Fig 5:**
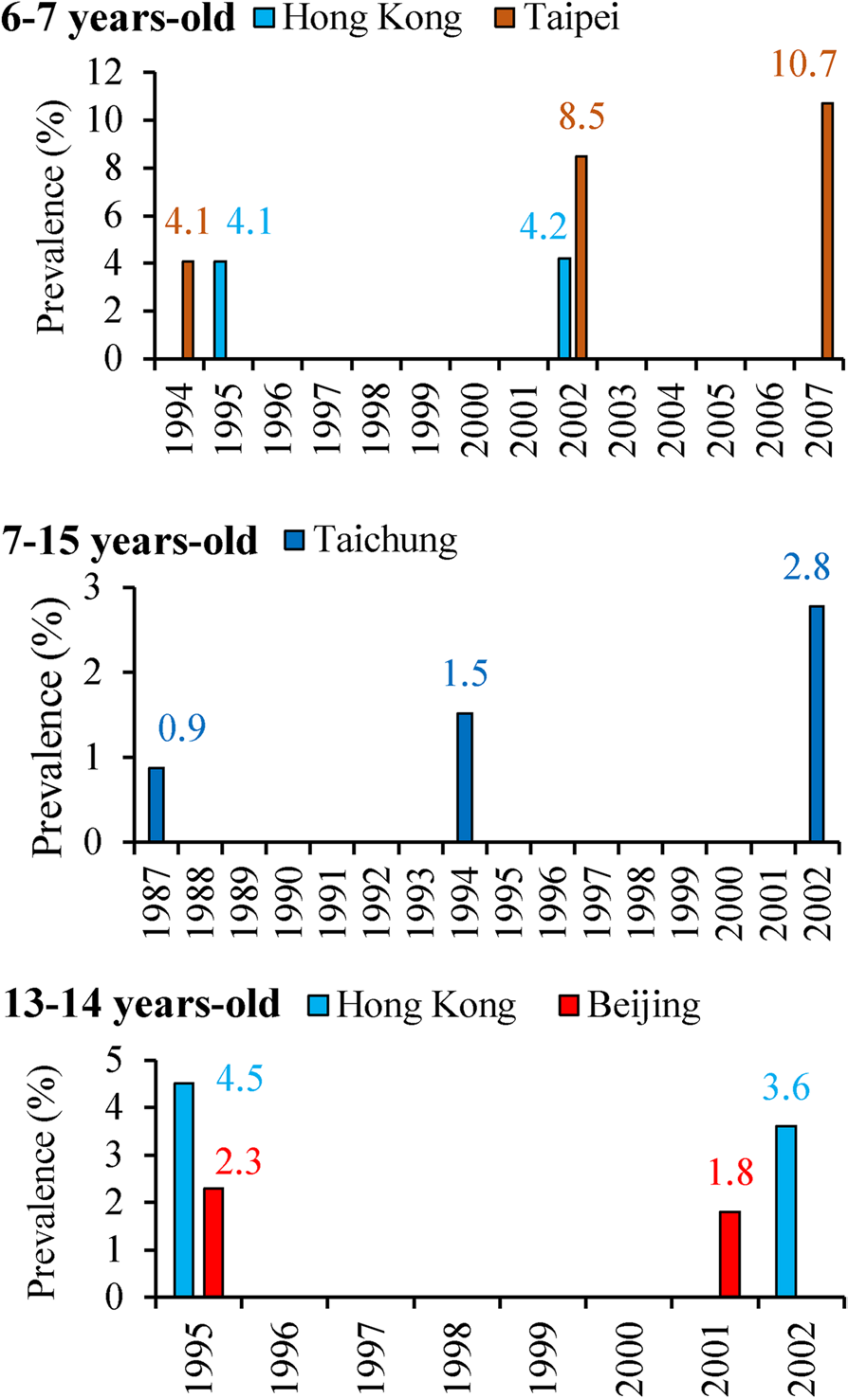
Time-trend of current (in the past 12 months prior to survey) eczema prevalences among children in specific age groups and cities. The number colors correspond to the bar colors for different cities

With regard to prevalences of current (in the past 12 months prior to survey) prior-year eczema in different age groups from ISAAC questionnaire-based cross-sectional studies (Fig. 4 and supplemental S1 Table), a total of 23 cities or regions (16 cities in mainland of China, 6 cities in Taiwan, and Hong Kong) had available data. Prevalences of prior-year eczema varied from 0.9% among 7-15-year-olds children in 1987 in Taichung, Taiwan [8] to 15.8% among 3-6-year-olds children in 2012 in Beijing [30], and mainly ranged from 4.0% to 15.0%. Increasing trends in eczema prevalences were found for most cities (Beijing, Urumqi, Xi’an, Shanghai, Chongqing, Wuhan, Guangzhou, Taipei, and Taichung). In those cities where data for prevalences of current eczema were available for more than two years (Fig 5), the eczema prevalences had increasing trends among children aged 6-7 and 7-15-year-olds, whereas prevalences were decreasing for 13-14-year-olds children from 1995 to 2001. In particular, eczema prevalences increased substantially for 6-7-year-olds children in Taipei from 4.1% in 1994 [9] to 8.5% in 2002 [22], and then to 10.7% in 2007 [22], as well as for 7-15-year-olds children in Taichung from 0.9% in 1987 [8] to 1.5% in 1994 [32], and then to 2.8% [38] in 2002 (Fig. 5).

**Fig 6:**
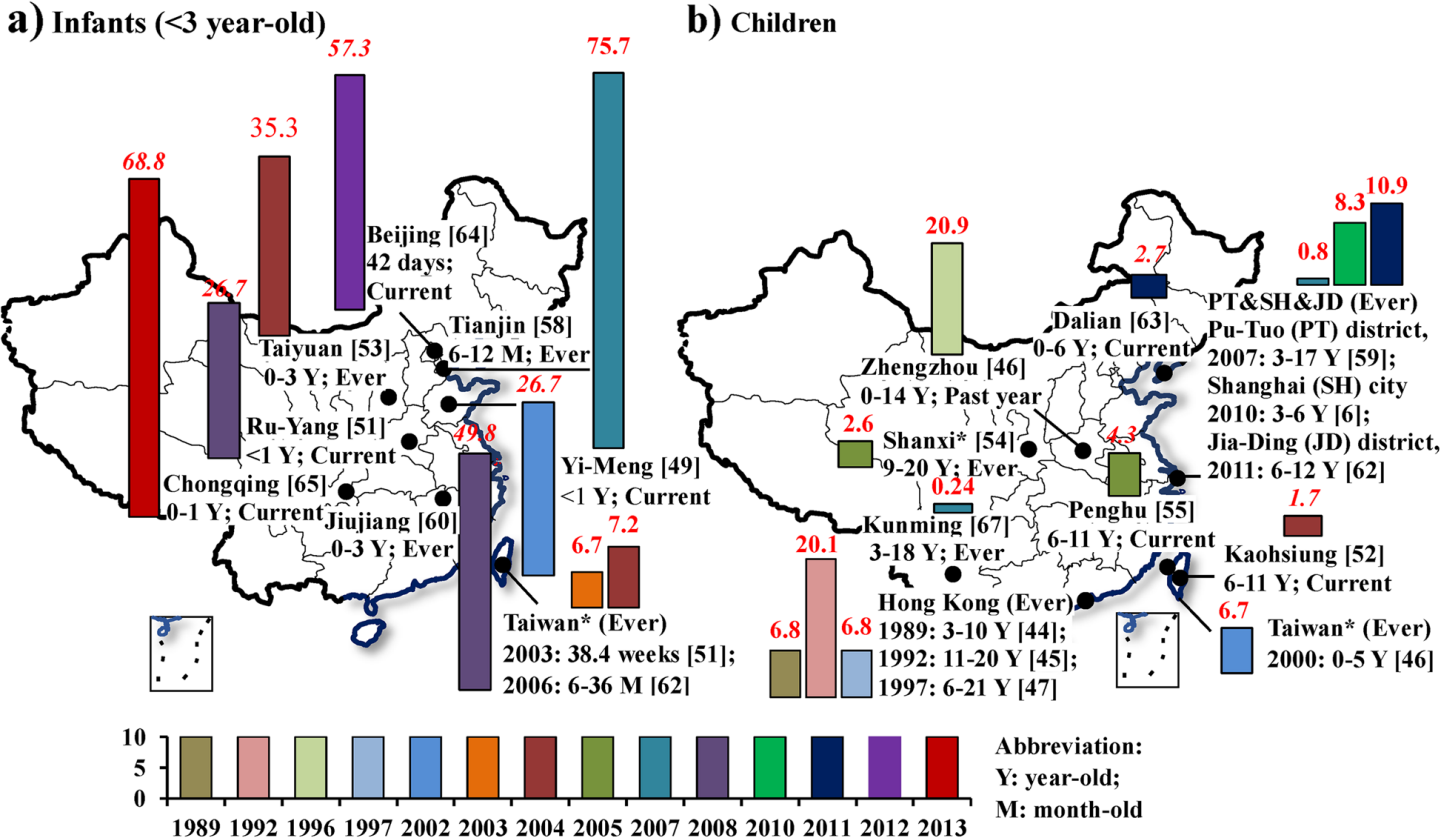
Summary of lifetime-ever and prior year eczema prevalences in children from studies which did not use the ISAAC questionnaire in China, 1985-2015. Herein, “*” indicates that the exact city where the study was conducted was not reported. The prevalences in *italic* indicates that the studied children were examined by dermatologists. The number in the bracket is the reference number. Supplemental S2 Table and S3 Table provide more information for these selected studies. The bar charts were conducted by Microsoft Excel 2013 and Word 2013 (https://www.microsoft.com/en-us/). The based maps of mainland China and Taiwan were generated by ESRI ArcGIS 10.0 version for desktop (http://www.esri.com/software/arcgis/arcgis-for-desktop)

In studies of children not using the ISAAC questionnaire (Fig. 6, supplemental S2 Table and S3 Table), a total of 16 cities had reported eczema prevalences. The highest eczema prevalence was 75.7% among 6-12-month-olds infants in 2007 in Tianjin [59], followed by 68.8% among 0-1-year-old infants in 2012 in Chongqing [50]. Eczema prevalences among infant groups were notably higher than among older children.

From the studies that provided eczema prevalences for boys and girls [7–10, 17, 18, 23, 26, 30, 31, 38, 39], boys’ prevalences were always higher than girls’ prevalences (supplemental S1-S3 Tables), except for Hong Kong [15, 17] and Beijing [18].

**Fig 7:**
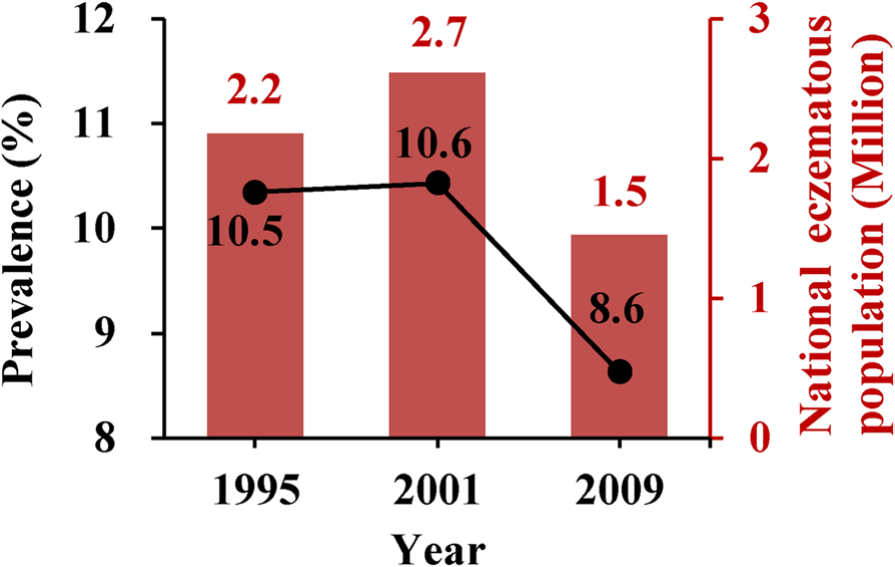
Time-trend of lifetime-ever eczema prevalence and eczematous populations among 13-14 year-old children in the mainland of China. For eczema prevalence in 1995, the surveyed cities were Beijing, Guangzhou, Urumqi, Shanghai, and Chongqing [16], and the national total population of 13-14 year-old children in 1996 was used to estimate the absolute eczematous population because data in 1995 were not available. For 2001, the surveyed cities were Beijing [24], Guangzhou [25], and Lhasa [26]. For 2009, the surveyed cities were Beijing, Guangzhou, and Chongqing [7]. More information for these studies is provided in Table 1

With regard to the national time-trend of eczema prevalence in mainland China, only data for children aged 13-14-year-olds during lifetime-ever were available (Fig. 7). The national-averaged lifetime-ever prevalence of eczema among 13-14-year-olds children slightly increased from 1995 to 2001, but notably decreased from 2001 to 2009. Similar trends were found for the absolute numbers of eczematous population among 13-14-year-olds children from 1995 to 2009. Table 2 shows national-averaged prevalences of eczema during different years and shows absolute numbers of eczematous populations among children in three age groups in mainland China. We estimated that 25.7% of children aged 3-6-year-olds or about 15.5 million had lifetime-ever eczema in 2012 in mainland China.

**Table 2:** National averaged prevalence of eczema and absolute eczematous populations among children in different age groups in mainland China

Year	Age (year-old)	Prevalence (%)	Population (million)	Reference
2005	6-13	Past year: 5.5	Past year: 8.2	[35]
2009	0-14	Ever: 14.5	Ever: 32.3	[7]
2012	3-6	Ever: 25.7; Past year: 12.2	Ever: 15.5; Past year: 7.4	[5]

### Adulthood eczema

**Fig 8:**
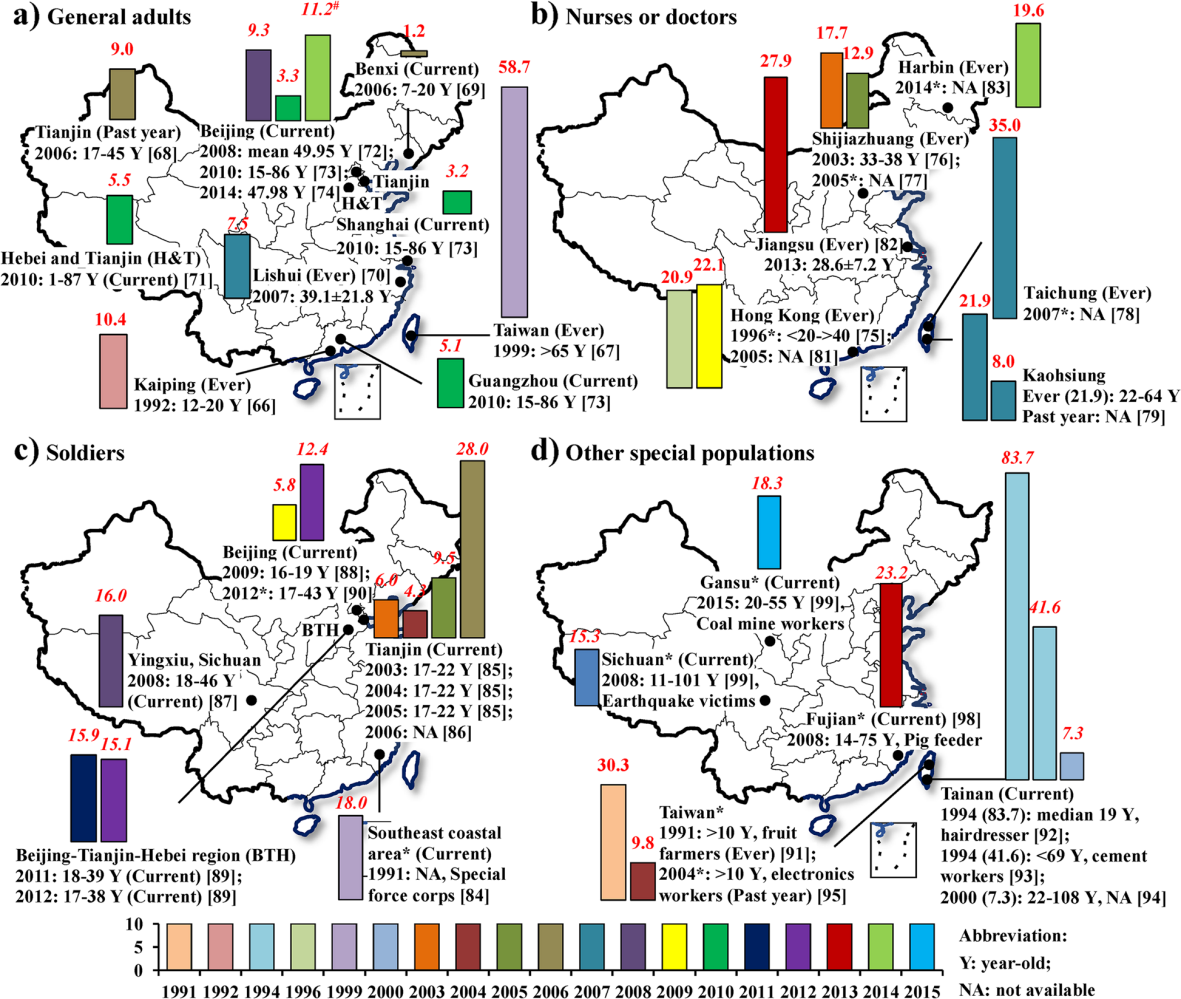
Summary of eczema prevalence during lifetime-ever or in the past year from studies of adults in China, 1985-2015. Herein “*” indicates that the exact city where the study was conducted or the exact year when the study was conducted was not provided in the literature; “^#^” indicates that the presented prevalence was averaged among prevalences for various age groups from different studies in the same year. The prevalences in *italic* indicates that the studied populations were examined by dermatologists. Number in the bracket is the reference. Supplemental S4-S7 Tables show more information for these selected studies. The bar charts were conducted by Microsoft Excel 2013 and Word 2013 (https://www.microsoft.com/en-us/). The based maps of mainland China and Taiwan were generated by ESRI ArcGIS 10.0 version for desktop (http://www.esri.com/software/arcgis/arcgis-for-desktop)

Figure 8 and supplemental S4-S7 Tables summarize prevalences of eczema of adults in studies from 1985 to 2015. Studies were limited and the studied subjects were so varied that time-trends could not be established. With regard to overall adult populations (Fig. 8a and supplemental S4 Table), a total of nine cities had available data. Eczema prevalences were notably different in different cities, varying from 1.2% for current eczema among 7-20-year-old people in Benxi, Liaoning in 2006 [70] to 58.7% for life-time ever eczema in >65-year-old Taiwan adults in 1999 [68]. Among studies which provided eczema prevalences for males and females [68, 70, 72, 73], males’ prevalences were higher than females’ prevalences (supplemental S4 Table) except for in Hebei and Tianjin among 1-87-year-olds people [72].

Six cities had data for eczema prevalences among nurses and/or doctors (Fig. 8b and supplemental S5 Table), ranging from 8.0% to 35.0%. Five cities or regions had data for eczema prevalences among soldiers (Fig. 8c and supplemental S6 Table) and other special populations (Fig. 8d and supplemental S7 Table). Prevalences among soldiers ranged from 4.3% to 28.0%. The highest prevalence among other special populations was 83.7% for current eczema among hairdresser (median 19-year-olds) in Tainan, Taiwan [93].

With regard to national-averaged prevalences of eczema and absolute eczema population among adults in the mainland of China (Table 3), we estimated that about 39.4, 20.0, and 11.6 million people among adults aged 15-86-year-olds had contact dermatitis, seborrheic dermatitis, and atopic dermatitis in 2010.

**Table 3:** National averaged prevalence of current dermatitis and absolute illnesses populations among general adults in mainland China

Year	Age (year-old)	Type of dermatitis	Prevalence (%)	Population (million)	Reference
2010	15-86	Contact dermatitis	3.5	39.4	[74]
		Seborrheic dermatitis	1.8	20.0	
		Atopic dermatitis	1.0	11.6	

## Discussion

In this review, we have summarized all studies of eczema prevalence among children and adults conducted in China from 1985 to 2015. Among children, there was an increasing trend in most cities. However, prevalence of lifetime-ever eczema and the eczematous population among children aged 13-14-year-olds decreased from 2001 to 2009. Limited data prevented us from discerning time-trends among adults. In general, eczema prevalences in younger children were higher than in older children, and in boys compared to girls, as well as in male adults compared to female adults.

Our findings that childhood eczema prevalences increased among 6-7-year-olds children but decreased among 13-14-year-olds children were partly consistent with those findings from the ISAAC study [2] and with findings of a systematic review of worldwide incidence and prevalence of atopic eczema [4]. Williams et al. found that eczema symptom prevalence decreased from 1995 to 2006 in some previously high prevalence centers in the developed world, whereas prevalences in developing countries with previously low prevalences continued to increase in 13-14-year-olds children, whereas most centers showed increasing prevalences in 6-7-year-olds children [2]. Deckers et al. found that eczema prevalences were increasing in Africa, eastern Asia, western Europe and parts of northern Europe from 1990 to 2010 [4]. The latest lifetime-ever eczema prevalences among 3-6-year-olds children in Beijing and Chongqing in 2012, as well as among 6-7-year-olds in Hong Kong in 2002 and in Taipei in 2007, ranged from 25.0%-35.0% (Fig. 3), higher than the global-averaged level among 6-7-year-olds children from the ISAAC study Phase Three in 2006 and were comparable to the high prevalences in developed countries reported in recent years [3].

Interestingly, we found that the national-averaged prevalence and absolute eczematous population of 13-14-year-olds children in mainland China decreased from 2001 to 2009. We suspect this may be related to the marked improvement in medical conditions and health services in mainland China in the past decades, as well as to the access to basic public health services has become more and more equitable [102]. Those children who were diagnosed with eczema at a younger age may have had better medical care in recent years than before 2001, such that when they reached 13-14-year-olds, eczema prevalences were lower than in 2009. This hypothesis also is supported by our finding that eczema prevalences among younger children substantially increased during the same years, whereas eczema prevalences among 13-14-year-olds children decreased in all specific cities (Figs. 3 and 5). In the present study, we also found that national absolute eczematous populations among young infants/children were substantially higher than in older children. This finding is consistent with several previous systematic reviews or individual studies, which have reported that atopic eczema was more common among younger infants/children than among older children [1, 14, 103–108]. Specifically, Kelbore et al. conducted a facility-based cross-sectional study among 477 children aged from three months to 14 years in the Ayder referral hospital in Mekelle, Ethiopia and found that children aged three months to one year had significantly higher risk of atopic dermatitis than older children (odds ratio: 6.8; 95 % confidence interval: 1.1-46.0) [105]. Hong et al. conducted an ISAAC questionnaire-based cross-sectional study among 31,201 Korean children and found that the past 12 months prevalences of dermatitis symptoms among 0-3, 4-6, 7-9, and 10-13-year-olds children were 19.3%, 19.7%, 16.7%, and 14.5%, respectively (*p*-value for trend <0.001) [108]. In general, eczema is considered to be an early infancy illness that will get better as infants grow older [106, 107]. However, other allergic diseases and/or symptoms are likely to appear in those children who had eczema as infants [109–112]. Thus, we consider it reasonable that older children had lower prevalences of eczema than young infants since eczema will get better as children grow older [112]. Also, prevalence of eczema among old children (13-14-year-olds) could have decreased trends if the medical conditions and health services improved [102].

There were too few studies of adults to discern time-trends or to compare eczema prevalences in different cities (Fig. 8). There was only one cross-sectional study in three large cities of eczema prevalence among an overall population of adults [74]. However, some studies reported high prevalences among several special populations, for example, nurses and/or doctors [76, 79, 80, 82], soldiers [85–90], fruit farmers [92], hairdressers [93], and cement workers [94]. These findings indicate certain populations with special occupations and with particular environmental exposures could had higher likelihood in getting eczema, and these exposures possibly are risk factors for eczema. These findings also indicate that the national burden of eczema in adults, especially among several special populations, could still be heavy even though the medical conditions and living conditions were improved in the past years in China [102].

Our finding that boys had higher eczema prevalences than girls in most cities is consistent with findings in several studies of young children [4, 107, 113–115] and adults [4, 106, 107]. However, our findings that male adults generally had higher eczema prevalences than female adults are inconsistent with findings from several previous studies showing that girls’ eczema prevalence catch up to boys’ prevalence around puberty [116–118]. Although these inconsistences could be due to the lack of data, more studies are necessary to clarify adult eczema prevalence in China [117, 118].

There are several gaps in the literature, especially for adults. Firstly, most of the information on eczema we collected was based on questionnaires, and the eczema questions differed, especially in those studies that did not use ISAAC questions. Symptoms of atopic eczema overlap with symptoms of other conditions, such as contact dermatitis [4]. In ISAAC-related questionnaire-based studies for children [5, 7–11, 15–44], childhood eczema was defined as an itchy rash coming and going for at least 6 months [3]. Several studies not using the ISAAC question [6, 62–64] used the UK diagnostic criteria [119] of atopic eczema: history of itchy skin plus at least three following signs: 1) history of rash in the skin creases (folds of elbows, behind the knees, fronts of ankles or around the neck); 2) history of asthma or hay fever: 3) history of dry skin in the last year; 4) onset under the age of 2-year-olds; and 5) visible flexural dermatitis as defined by a photographic protocol [120]. Chinese non-ISAAC studies [50, 52, 66] based a clinical diagnosis of eczema on definitions from two Chinese textbooks (Clinical Dermatology; Practical Pediatric Dermatology) [121, 122] and a Chinese guidebook for eczema diagnosis [123]. In these Chinese books, the definition of eczema is: 1) mild level: skin lesion and erythema; 2) moderate level: papule or cracked skin and desquamation; 3) severe level: with blister, erosion and scabs. The Chinese criteria are less specific than the UK diagnostic criteria [119]. Nonetheless, both the UK diagnostic criteria [119] and the Chinese criteria [121–123] are stricter that in the ISAAC questionnaire [3]. Studies of adults used different clinical diagnostic criteria: two studies [73, 75] used the definition of eczema in “Clinical Dermatology” [121] and one study [72] used the UK diagnostic criteria of atopic eczema [119]. Secondly, ages of both children and adult varied in the available different studies (supplemental S1-S7 Tables). Eczema prevalence varies with age in both children and adults [14, 103–105]. Take the ISAAC-based studies in Beijing as an example (Fig. 2), logically, prevalences of eczema among children could not sharply decrease within one year. Since the definition of eczema and method for survey in these studied are the same, the main reason for these illogical trends of eczema prevalences probably is that ages of the children are largely different among these studies: the 10.3% in 1995 was among 6-7-year-olds children [5] and the 3.8% in 1996 was among 13-14 year-old children [7], as well as the 29.4% in 2008 was among 5-11-year-olds children [18] and the 20.6% in 2009 was among 0-14-year-olds children [24]. Thirdly, a large number of the selected studies did not provide sex information for eczema prevalence. Thus, we could not establish and compare the time-trends for boys and girls. Fourthly, most selected studies were conducted in large cities; there were few studies eczema in rural populations in mainland China. Several studies have reported that rural eczema prevalences are substantially lower than urban eczema prevalences [2, 4, 6, 40, 50, 118]. Thus, we have likely overestimated national eczema prevalence. Also, few studies have been done of non-Han Chinese, so we do not know the magnitude or direction of error that this lack of information introduces [3, 18, 26, 113].

To our best knowledge, no systematic review has summarized national time-trends of eczema prevalences or absolute eczematous populations of children and adults in China. The 93 articles [5–11, 15–100] we summarized in the quantitative synthesis had similar findings regarding eczema prevalences and their time-trends, and so can be said to give a reasonably accurate picture of eczema from 1985 to 2015 in China. Two reviewers independently checked the quality of all 93 full articles [5–11, 15–100] with consideration of the definition of eczema, the studied locations and populations, as well as the method used for sample and survey. Although there were only 16 articles [5, 7–9, 11, 15–18, 24–27, 29, 35, 44] for children and for young children, and only one for adults [74] that used the ISAAC questionnaire, most of these articles had large sample sizes and high response rates (Table 1). The ISAAC questions have been shown to provide adequate symptom-derived estimates of eczema prevalence [124, 125]. Another study has shown that the Chinese translated version of the ISAAC core questions provides sufficient information for atopic eczema among children in China [126]. Thus, the ISAAC questionnaire studies likely have high reliability with respect to eczema prevalence. The quantitative synthesis for the national-levels eczema prevalences used only those studies of at least three cities for mainland China [5, 7, 16, 35, 74] and therefore we considered those estimated national prevalence of eczema and absolute eczematous populations could indicate the actual status, at least in cities of mainland China.

In future work, the diagnostic criteria for atopic eczema among infants, children, and adults should be standardized, and the questionnaire designed according to these standards. Since the effectiveness of ISAAC core questions for childhood eczema have been validated [124–126], we recommend using this questionnaire for eczema [5]. Nation-wide studies stratified by age-groups and sex, and with standardized reporting format would be useful to compare prevalences and time-trends in cities.

Many studies have tried to find explanations for the changing time-trends in eczema prevalences among children and adults [1, 4, 127–139]. The prevalence changes could be an artifact of methodology, due to possible changes in diagnostic criteria over time, and differences in the study design [4, 140–142]. However, the prevalence changes could be real. Several studies have proposed that changes in household and ambient environment-related exposures [127–134, 139], while others have suggested changes in lifestyle and dietary habits [135–138], may be causing increased eczema prevalences. Specifically, several studies have presented evidence that indoor air pollution [127, 128] and home dampness-related exposures [69, 130–134, 139] are risk factors for childhood and adult eczema. Outdoor air pollution [129], shorter breastfeeding [133], antibiotic use in infancy [135], early pet-keeping [137], and eating fast foods [136, 138] have been suspected of association with childhood eczema, but findings from different studies have been inconsistent regarding these factors. More well-designed and large-scale studies are warranted to provide explanations for the increase in eczema prevalence among children and adults in China.

This study had some limitations. First, the collected eczema prevalences among children were based on the population-based survey and parents-reported or self-reported questionnaire. The studied eczema was defined using a positive answer to a single question in the most studies that were based on the ISAAC questionnaire. There may be reporting error and recall bias. Second, the definition in different studies could have notable differences, especially for those studies among adults that were not based on the ISAAC questionnaire. Third, in the estimation of absolute eczematous populations, we assumed that the proportions of populations for each specific age were the same within various age groups. This assumption could introduce error in the estimation.

## Conclusions

Eczema prevalences among 3-12-year-olds children have increased in most Chinese cities during 1985 to 2015. From 2001 to 2009, lifetime-ever eczema prevalence and the absolute eczematous population among children aged 13-14-year-olds decreased. We estimate that about 1.5 million 13-14-year-olds children in 2009 and about 15.5 million 3-6-year-olds children in 2012 had lifetime-ever eczema in mainland China. We estimated that approximately 39.4 (prevalence: 3.5%), 20.0 (1.8%), and 11.6 (1.0%) million people among 15-86-year-olds adults in 2010 had contact dermatitis, seborrheic dermatitis, and atopic dermatitis, respectively. The national burden of eczema became heavier among young children, whereas perhaps had been reduced among adolescent. More studies for adults are warranted to ascertain time-trends of eczema prevalence among adults.

## Supplementary Information

**Additional file 1.**

## Data Availability

All data generated or analyzed during this study are included in this published article and its supplementary information files.

## References

[1] Weidinger S, Novak N (2016). Atopic dermatitis. Lancet.

[2] Williams H, Stewart A, von Mutius E, Cookson W, Anderson HR (2008). ISAAC Phase One Three Study Grp. Is eczema really on the increase worldwide?. J. Allergy Clin. Immunol..

[3] Asher MI (2006). Worldwide time trends in the prevalence of symptoms of asthma, allergic rhinoconjunctivitis, and eczema in childhood: ISAAC Phases One and Three repeat multicountry cross-sectional surveys. Lancet.

[4] Deckers IA (2012). Investigating international time trends in the incidence and prevalence of atopic eczema 1990-2010: a systematic review of epidemiological studies. PLoS One.

[5] Zhang YP (2013). Ten cities cross-sectional questionnaire survey of children asthma and other allergies in China. Chin. Sci. Bull..

[6] Xu F (2012). Prevalence of childhood atopic dermatitis: an urban and rural community-based study in Shanghai, China. PLoS One.

[7] Zhao J (2010). Self-reported prevalence of childhood allergic diseases in three cities of China: a multicenter study. BMC Public Health.

[8] Liao PF, Sun HL, Lu KH, Lue KH (2009). Prevalence of childhood allergic diseases in Central Taiwan over the past 15 years. Pediatr. Neonatol..

[9] Wu WF, Wan KS, Wang SJ, Yang W, Liu WL (2011). Prevalence, severity, and time trends of allergic conditions in 6-to-7-year-old schoolchildren in Taipei. J. Invest. Allergy Clin..

[10] Song N (2014). Prevalence, severity and risk factors of asthma, rhinitis and eczema in a large group of Chinese schoolchildren. J. Asthma.

[11] Lee YL, Li CW, Sung FC, Guo YL (2007). Increasing prevalence of atopic eczema in Taiwanese adolescents from 1995 to 2001. Clin. Exp. Allergy.

[12] Asher MI (1995). International Study of Asthma and Allergies in Childhood (ISAAC): rationale and methods. Eur. Respir. J..

[13] Natural Bureau of Statistics of the People’s Republic of China. China Statistical Yearbook (1997-2015). http://www.stats.gov.cn/tjsj/ndsj/. Accessed 1 Apr 2016.

[14] Huang C (2015). Updated prevalences of asthma, allergy, and airway symptoms, and a systematic review of trends over time for childhood asthma in Shanghai, China. PLoS One.

[15] Leung R (1997). Prevalence of asthma and allergy in Hong Kong schoolchildren: an ISAAC study. Eur. Respir. J..

[16] Chen YZ (1998). A questionnaire-based survey on prevalences of asthma, allergic rhinitis and eczema in five Chinese cities. Chin. J. Pediatr..

[17] Lau YL, Karlberg J (1998). Prevalence and risk factors of childhood asthma, rhinitis and eczema in Hong Kong. J. Paediatr. Child Health.

[18] Zhao TB (2000). Prevalence of childhood asthma, allergic rhinitis and eczema in Urumqi and Beijing. J. Paediatr. Child Health.

[19] Lee YL (2003). Climate, traffic-related air pollutants and allergic rhinitis prevalence in middle-school children in Taiwan. Eur. Respir. J..

[20] Wong GWK (2001). Prevalence of respiratory and atopic disorders in Chinese schoolchildren. Clin. Exp. Allergy.

[21] Wang WC, Lue KH, Sheu JN (1998). Allergic diseases in preschool children in Taichung City. Acta Paediatrica Sinica.

[22] Chen CF, Wu KG, Hsu MC, Tang RB (2001). Prevalence and relationship between allergic diseases and infectious diseases. J. Microbiol. Immunol. Infect..

[23] Lee YL (2007). Environmental factors, parental atopy and atopic eczema in primary-school children: a cross-sectional study in Taiwan. Brit. J. Dermatol..

[24] Ma Y, Kang XH, Zhang JL (2004). Prevalence of asthmatic and atopic disorders in Chinese schoolchildren in Beijing, a comparison between 2001 and 1994. Beijing Med. J..

[25] Wang HY, Zheng JP, Zhong NS (2006). Time trends in the prevalence of asthma and allergic diseases over 7 years among adolescents in Guangzhou city. Zhonghua Yi Xue Za Zhi.

[26] Droma Y (2007). Prevalence and severity of asthma and allergies in schoolchildren in Lhasa, Tibet. Clin. Exp. Allergy.

[27] Lee SL, Wong W, Lau YL (2004). Increasing prevalence of allergic rhinitis but not asthma among children in Hong Kong from 1995 to 2001 (Phase 3 International Study of Asthma and Allergies in Childhood). Pediatr. Allergy Immunol..

[28] Chen WY (2003). Synergistic effect of multiple indoor allergen sources on atopic symptoms in primary school children. Environ. Res..

[29] Wong GWK (2004). Declining asthma prevalence in Hong Kong Chinese schoolchildren. Clin. Exp. Allergy.

[30] Kao CC, Huang JL, Ou LS, See LC (2005). The prevalence, severity and seasonal variations of asthma, rhinitis and eczema in Taiwanese schoolchildren. Pediatr. Allergy Immunol..

[31] Liao MF, Huang JL, Chiang LC, Wang FY, Chen CY (2005). Prevalence of asthma, rhinitis, and eczema from ISAAC survey of schoolchildren in central Taiwan. J. Asthma.

[32] Chiang LC, Chen YH, Hsueh KC, Huang JL (2007). Prevalence and severity of symptoms of asthma, allergic rhinitis, and eczema in 10-to 15-year-old schoolchildren in central Taiwan. Asian Pac. J. Allergy.

[33] Zeng SW, Tang NJ, Ji LM, Coenraads PJ (2005). Prevalence of atopic dermatitis in infants and children in Tianjin, China. J. Invest. Dermatol..

[34] Wu WC (2011). Psychosocial problems in children with allergic diseases: a population study in Taiwan. Child Care Health Dev..

[35] Li F (2011). Prevalence and risk factors of childhood allergic diseases in eight metropolitan cities in China: A multicenter study. BMC Public Health.

[36] Hsu NY, Wu PC, Bornehag CG, Sundell J, Su HJ (2012). Feeding bottles usage and the prevalence of childhood allergy and asthma. Clin. Dev. Immunol..

[37] Lee SL (2012). Foetal exposure to maternal passive smoking is associated with childhood asthma, allergic rhinitis, and eczema. Scientific World J..

[38] Liao MF, Liao MN, Lin SN, Chen JY, Huang JL (2009). Prevalence of allergic diseases of schoolchildren in Central Taiwan. J. Asthma.

[39] Yao TC (2011). Associations of age, gender, and BMI with prevalence of allergic diseases in children: PATCH study. J. Asthma.

[40] Lv HB, Deng FR, Sun JD (2010). The comparison of the indoor environmental factors associated with asthma and related allergies among school-child between urban and suburban area in Beijing. Chin. J. Prev. Med..

[41] Wang IJ (2013). Maternal employment and atopic dermatitis in children: a prospective cohort study. Br. J. Dermatol..

[42] Wang J (2014). Epidemiological survey on bronchial asthma, allergic rhinitis and eczema among urban children in Guangxi. Mat. Child Health Care China.

[43] Lin MH (2015). Fetal growth, obesity, and atopic disorders in adolescence: a retrospective birth cohort study. Paediatr. Perinat. Epidemiol..

[44] Yang Z (2015). Frequency of food group consumption and risk of allergic disease and sensitization in schoolchildren in urban and rural China. Clin. Exp. Allergy.

[45] Lau YL, Karlberg J, Yeung CY (1995). Prevalence of and factors associated with childhood asthma in Hong Kong. Acta Paediatr.

[46] Leung R, Ho P (1994). Asthma, allergy, and atopy in three south-east Asian populations. Thorax.

[47] Chen LL, Ma F, Han FG (1997). Investigation and analysis of otolaryngology diseases among 4154 children in Zhengzhou. Henan J. Pre. Med..

[48] Fung WK, Lo KK (2000). Prevalence of skin disease among school children and adolescents in a Student Health Service Center in Hong Kong. Pediatr. Dermatol..

[49] Sun HL, Yeh CJ, Ku MS, Lue KH (2012). Coexistence of allergic diseases: patterns and frequencies. Allergy Asthma Proc..

[50] Wang LY, Wang HJ, Meng J, Zhang YX (2001). Investigation and analysis of infant eczema in Yi-Meng mountain area. Chin. J. Dermatol. Venereol..

[51] Wang IJ (2007). Environmental risk factors for early infantile atopic dermatitis. Pediatr. Allergy Immunol..

[52] Zhang ZK (2006). Investigation and analysis for the situation of infant eczema. J. Med. Forum.

[53] Yang YC, Cheng YW, Lai CS, Chen W (2007). Prevalence of childhood acne, ephelides, warts, atopic dermatitis, psoriasis, alopecia areata and keloid in Kaohsiung County, Taiwan: a community-based clinical survey. J. Eur. Acad. Dermatol..

[54] He YH, Kang J, Liu GZ, Chen GT (2006). Analysis of factors relevant to the onset of eczema in 479 infants in Taiyuan. Chin. J. Info. TCM..

[55] Norbäck D (2007). Asthma, eczema, and reports on pollen and cat allergy among pupils in Shanxi province, China. Int. Arch. Occup. Environ. Health.

[56] Chen GY (2008). Prevalence of skin diseases among schoolchildren in Magong, Penghu, Taiwan: A community-based clinical survey. J. Formos Med. Assoc..

[57] Huang CC (2015). Prenatal air pollutant exposure and occurrence of atopic dermatitis. Br. J. Dermatol..

[58] Wen HJ (2009). Predicting risk for early infantile atopic dermatitis by hereditary and environmental factors. Br. J. Dermatol..

[59] Liu J, Ye T, Li YM, Zhang LX, Xu BL (2008). Epidemiological investigation on infant eczema symptoms. Mat. Child Health Care China.

[60] Shi YM, Zhang FY, Dai HL, Zhang SZ, Hang JQ (2010). Prevalence of atopic disorders in school children in Changzheng town of Putuo district, Shanghai. Chin. J. General Practice.

[61] Hu HD (2010). Analysis of related factors for infant eczema among 500 cases. Chin. J. Mod. Drug Appl..

[62] Wang MH (2011). Investigation on childhood atopic dermatitis in Kunming. Chin. J. Public Health.

[63] Xu F (2012). Childhood atopic dermatitis and household environmental risk factors: a cross-sectional study in 4784 children in Jiading district, Shanghai. J. Environ. Health.

[64] Wei FL (2012). Epidemiological survey of atopic dermatitis among pre-school children in Dalian. Chin. J. Leprosy Skin Dis..

[65] Shen CP (2015). Epidemiologic investigation and treatment for the younger infantile eczema. J. Clin. Dermatol..

[66] Feng M, Xiao YZ, Luo XY, Hu Y (2015). Investigations of environmental risk factors of eczema in different genetic background infants. Chin. J. Child Health Care.

[67] Leung R, Jenkins M (1994). Asthma, allergy and atopy in southern Chinese school students. Clin. Exp. Allergy.

[68] Liao YH, Chen KH, Tseng MP, Sun CC (2001). Pattern of skin diseases in a geriatric patient group in Taiwan: A 7-year survey from the outpatient clinic of a University Medical Center. Dermatology.

[69] Sun Y, Zhang Y, Sundell J, Fan Z, Bao L (2009). Dampness at dorm and its associations with allergy and airways infection among college students in China: a cross-sectional study. Indoor Air.

[70] Zhao LP (2013). Epidemiological survey of atopic dermatitis in youngsters in Benxi. Chin. J. Dermatol. Venereol..

[71] Lu XY, Li LF, You YM (2008). Prevalence of and risk factors for skin diseases in a community of Lishui City. China J. Lepr. Skin Dis..

[72] Wang ZH (2012). Analysis of the correlation of prevalence in allergic rhinitis and other allergic diseases. Chin. J. Otorhinolaryngol. Head Neck Surg..

[73] You YM, Li LF (2011). The prevalence of skin diseases in a community of Beijing and analysis of risk factors. Chin. J. Dermatol. Venereol..

[74] Xu F (2013). Self-declared sensitive skin in China: a community-based study in three top metropolises. J. Eur. Acad. Dermatol..

[75] Li J, You YM, Feng H (2015). Prevalence of chronic eczema in urban and urban fringe of one district in Beijing and analysis of risk factors. China J. Modern Med..

[76] Leung R, Ho A, Chan J, Choy D, Lai CKW (1997). Prevalence of latex allergy in hospital staff in Hong Kong. Clin. Exp. Allergy.

[77] Smith DR, Wei N, Zhao L, Wang RS (2005). Hand dermatitis among nurses in a newly developing region of Mainland China. Int. J. Nurs. Study.

[78] Smith DR, Wei N, Zhang YJ, Wang RS (2005). Hand dermatitis among a complete cross-section of Chinese physicians. Contact Dermatitis.

[79] Lin CT (2008). A hospital-based screening study of latex allergy and latex sensitization among medical workers in Taiwan. J. Microbiol. Immunol..

[80] Lan CCE (2008). Hand eczema among University Hospital nursing staff: identification of high-risk sector and impact on quality of life. Contact Dermatitis.

[81] Lan CCE (2009). Prevalence of adult atopic dermatitis among nursing staff in a Taiwanese medical center: a pilot study on validation of diagnostic questionnaires. J. Am. Acad. Dermatol..

[82] Luk NMT (2011). Hand eczema among Hong Kong nurses: a self-report questionnaire survey conducted in a regional hospital. Contact Dermatitis.

[83] Liu LP, Li YM, Xu H, Ma H (2014). Self-administered questionnaire on hand eczema in nurses. J. Clin. Dermatol..

[84] Zhang D (2014). Correlation analysis on the pathogenic factors for nurse hand eczema. Latest Med. Info. Essay (Electronic edition).

[85] Che DF (2001). Investigation and prophylaxis of the high-morbidity dermatoses in a certain special army unit in the southeast of China. J. Med. Postgraduates.

[86] Niu YT (2007). Relationship between military training and dermatitis in armed police forces soldiers. Med. J. Chin. People’s Armed Police Forces.

[87] Lu, T., Niu, Y.T., Xu, H.H., Lin, C.L., Wang, W. An investigation and analysis of 3748 military medical fee-free out-patients in a troops hospital dermatological department. Acta Acad. Med. CPAF 20(9), 692-693+713 (2011). (In Chinese)

[88] Shi YX (2009). Analyze traumas and diseases in part of the soldiers of the earthquake-rescue troops in Yingxiu town and our experience on treatment and prevention. J. Navy Med..

[89] Chen WH, Li XP, Gu XF, Liu J, Xu DH (2011). Investigation on skin disease among Navy flying cadets before and after training. Military Med. J. Southeast China.

[90] Tian YL, Wang WL, Song KM, Su YM, Peng SW (2012). Analysis on the incidence and distribution of dermatoses among officers and soldiers in area of Beijing, Tianjin and Hebei Province. J. Pract. Dermatol..

[91] Zhu H, Yang RY (2013). Dermatopathic investigation of Northern China pontoon bridge troop in the summer. J. Pract. Dermatol..

[92] Guo YL, Wang BJ, Lee CC, Wang JD (1996). Prevalence of dermatoses and skin sensitisation associated with use of pesticides in fruit farmers of southern Taiwan. Occup. Environ. Med..

[93] Guo YL, Wang BJ, Lee JYY, Chou SY (1994). Occupational hand dermatoses of hairdressers in Tainan City. Occup. Environ. Med..

[94] Guo YL (1999). Dermatoses in cement workers in southern Taiwan. Contact Dermatitis.

[95] Smith DR (2002). Prevalence of skin disease among nursing home patients in southern Taiwan. Int. J. Dermatol..

[96] Shiao JSC, Sheu HM, Chen CJ, Tsai PJ, Guo YL (2004). Prevalence and risk factors of occupational hand dermatoses in electronics workers. Toxicol. Ind. Health.

[97] Nie YL (2009). Spectrum of diseases in 15 towns of earthquake attacked areas in Sichuan province. Med. J. Chin. PLA.

[98] Jia N (2012). Hand eczema and its risk factors of workers in ferrous metal smelting and Calendering processing industry. J. Environ. Occup. Med..

[99] Zhang YF (2014). Analysis of risk factors for contact dermatitis in pig farm workers. Chin. J. Ind. Hyg. Occup. Dis..

[100] Zhang, J.X., He, Y.Y. & Liu, H.Z. Investigation on the risk factors of eczema in coal mine workers. Chin. Community Doctors 23, 75-75+77 (2015). (In Chinese)

[101] Willis CM (2001). Sensitive skin: an epidemiological study. Br. J. Dermatol..

[102] Information Office of the State Council, the People's Republic of China. White Paper: Medical and Health Services in China. Beijing: Foreign Languages Press; 2012.

[103] Eichenfield LF (2014). Guidelines of care for the management of atopic dermatitis: Part 1: Diagnosis and Assessment of Atopic Dermatitis. J. Am. Acad. Dermatol..

[104] Oh JW (2004). Epidemiological change of atopic dermatitis and food allergy in school-aged children in Korea between 1995 and 2000. J. Korean Med. Sci..

[105] Kelbore AG, Alemu W, Shumye A, Getachew S (2015). Magnitude and associated factors of atopic dermatitis among children in Ayder referral hospital, Mekelle, Ethiopia. BMC Dermatol.

[106] Bieber T (2008). Atopic dermatitis. N. Engl. J. Med..

[107] Wallach D, Taïeb A (2014). Atopic dermatitis/Atopic eczema. Chem. Immunol. Allergy.

[108] Hong S (2012). The prevalence of atopic dermatitis, asthma, and allergic rhinitis and the comorbidity of allergic diseases in children. Environ. Health Toxicol..

[109] Gustafsson D, Sjöberg O, Foucard T (2000). Development of allergies and asthma in infants and young children with atopic dermatitis--a prospective follow-up to 7 years of age. Allergy.

[110] Ekbäck M (2014). Severe eczema in infancy can predict asthma development. A prospective study to the age of 10 years. PLoS One.

[111] von Kobyletzki LB (2012). Eczema in early childhood is strongly associated with the development of asthma and rhinitis in a prospective cohort. BMC Dermatol..

[112] Barnetson RS, Rogers M (2002). Childhood atopic eczema. Br. Med. J..

[113] Kim HB (2016). Lifetime prevalence of childhood eczema and the effect of indoor environmental factors: Analysis in Hispanic and non-Hispanic white children. Allergy Asthma Proc..

[114] Cramer C (2011). Association between attendance of day care centres and increased prevalence of eczema in the German birth cohort study LISAplus. Allergy.

[115] Schäfer T, Stieger B, Polzius R, Krauspe A (2008). Atopic eczema and indoor climate: results from the children from Lübeck allergy and environment study (KLAUS). Allergy.

[116] Gough H (2015). Allergic multimorbidity of asthma, rhinitis and eczema over 20 years in the German birth cohort MAS. Pediatr. Allergy Immunol..

[117] Kahwa EK (2012). Asthma and allergies in Jamaican children aged 2-17 years: a cross-sectional prevalence survey. BMJ Open.

[118] Montnemery P, Nihlén U, Löfdah CG, Nyberg P, Svensson Å (2003). Prevalence of self-reported eczema in relation to living environment, socio-economic status and respiratory symptoms assessed in a questionnaire study. BMC Dermatol..

[119] Williams HC, Burney PG, Pembroke AC, Hay RJ (1996). Validation of the UK diagnostic criteria of atopic dermatitis in a population setting. U.K. Diagnostic Criteria for Atopic Dermatitis Working Party. Br. J. Dermatol..

[120] Williams HC, Forsdyke H, Boodoo G (1995). A protocol for recording the sign of flexural dermatitis in children. Br. J. Dermatol..

[121] Zhao B (1989). Clinical Dermatology, Second version.

[122] Tu YY, Yuan CY (1986). Practical Pediatric Dermatology.

[123] Immune Group in the Skin Venereology Branch of Chinese Medical Association (2011). Chinese guidelines for eczema diagnosis. Chin. J. Dermatol.

[124] Flohr C (2009). How well do questionnaires perform compared with physical examination in detecting flexural eczema? Findings from the International Study of Asthma and Allergies in Childhood (ISAAC) Phase Two. Br. J. Dermatol..

[125] Brenninkmeijer EEA, Schram ME, Leeflang MMG, Bos JD, Spuls PI (2008). Diagnostic criteria for atopic dermatitis: a systematic review. Br. J. Dermatol..

[126] Chan HH, Pei A, Van Krevel C, Wong GWK, Lai CKW (2001). Validation of the Chinese translated version of ISAAC core questions for atopic eczema. Clin. Exp. Allergy.

[127] Kim EH (2015). Indoor air pollution aggravates symptoms of atopic dermatitis in children. PLoS One.

[128] Cabieses B, Uphoff E, Pinart M, Antó JM, Wright J (2014). A systematic review on the development of asthma and allergic diseases in relation to international immigration: the leading role of the environment confirmed. PLoS One.

[129] Anderson HR (2010). Ambient particulate pollution and the world-wide prevalence of asthma, rhinoconjunctivitis and eczema in children: Phase One of the International Study of Asthma and Allergies in Childhood (ISAAC). Occup. Environ. Med..

[130] Ukawa S, Araki A, Kanazawa A, Yuasa M, Kishi R (2014). The relationship between atopic dermatitis and indoor environmental factors: a cross-sectional study among Japanese elementary school children. Int. Arch. Occup. Environ. Health.

[131] Bornehag CG (2005). ‘Dampness’ at home and its association with airway, nose, and skin symptoms among 10,851 preschool children in Sweden: a cross-sectional study. Indoor Air.

[132] Cai J (2016). Associations between home dampness-related exposures and childhood eczema among 13,335 preschool children in Shanghai, China: A cross-sectional study. Environ. Res..

[133] Clayton T (2013). Time trends, ethnicity and risk factors for eczema in New Zealand children: ISAAC Phase Three. Asia Pac. Allergy.

[134] Tsakok T, et al. Eczema and indoor environment: lessons from the International Study of Asthma and Allergies in Childhood (ISAAC) Phase 2. Lancet. 2015;385(99) poster 43. 10.1016/S0140-6736(15)60414-7 26312922

[135] Foliaki S, Pearce N, Björkstén B (2009). Antibiotic use in infancy and symptoms of asthma, rhinoconjunctivitis, and eczema in children 6 and 7 years old: International Study of Asthma and Allergies in Childhood Phase III. J. Allergy Clin. Immunol..

[136] Ezzati M, Riboli E (2013). Behavioral and dietary risk factors for noncommunicable diseases. N. Engl. J. Med..

[137] Huang C, Hu Y, Liu W, Zou ZJ, Sundell J (2013). Pet-keeping and its impact on asthma and allergies among preschool children in Shanghai. China. Chin. Sci. Bull..

[138] Tilman D, Clark M (2014). Global diets link environmental sustainability and human health. Nature.

[139] Mendell MJ, Mirer AG, Cheung K, Tong M, Douwes J (2011). Respiratory and allergic health effects of dampness, mold, and dampness-related agents: a review of the epidemiologic evidence. Environ. Health Perspect..

[140] Liu P (2016). Clinical features of adult/adolescent atopic dermatitis and Chinese criteria for atopic dermatitis. Chin. Med. J..

[141] Schmitt J (2013). Assessment of clinical signs of atopic dermatitis: A systematic review and recommendation. J. Allergy Clin. Immunol..

[142] Darsow U (2013). Difficult to control atopic dermatitis. World Allergy Organ. J..

