# Supplementary material for: Time-trends for eczema prevalences among children and adults from 1985 to 2015 in China: a systematic review
Source: BMC Public Health. 2022 Jul 5;22:1294. doi: 10.1186/s12889-022-13650-7 (PMC9254617; doi:10.1186/s12889-022-13650-7)
Supplement: Supplementary file 1 — Additional file 1. [file 12889_2022_13650_MOESM1_ESM.docx]

**Supplemental Information for**

***Time-Trends for Eczema Prevalences among Children and Adults from 1985 to 2015 in China: A Systematic Review***

Wei Liu^1^, Jiao Cai^1^, Chanjuan Sun^2^, Zhijun Zou^2^, Jialing Zhang^2^, Chen Huang^2,^ *

^1^ Institute for Health and Environment, Chongqing University of Science and Technology, Chongqing, China; ^2^School of Environment and Architecture, University of Shanghai for Science and Technology, Shanghai, China

* Corresponding Author:

Prof. Chen Huang

E-mail: huangc@usst.edu.cn; hcyhyywj@163.com

School of Environment and Architecture,

University of Shanghai for Science and Technology

516 Jungong Road, Yangpu District, Shanghai, PR China.

Tel: 021-55273409; Fax: 021-55270680

**Content**

**S1 Table.** Basic information for studies based on the ISAAC questionnaire in China, 1985-2015.

**S2 Table**. Basic information for studies that were not based on the ISAAC questionnaire for infants, 1985-2015.

**S3 Table**. Basic information for studies that were not based on the ISAAC questionnaire for preschool and school children, 1985-2015.

**S4 Table**. Basic information for studies which of eczema prevalence among common adult populations.

**S5 Table**. Studies of eczema prevalence in nurses and/or doctors in China.

**S6 Table**. Studies of eczema prevalence in soldiers in China.

**S7 Table**. Studies of eczema prevalence in other special populations in China.

**S1 Table.** Basic information for studies based on the ISAAC questionnaire in China, 1985-2015.

| Year ^a^ [Reference] | Age  (years) | City | Location and Inspection method | Sample size (response rate) | Prevalence, *N* (%) or % | | |
| --- | --- | --- | --- | --- | --- | --- | --- |
|  |  |  |  |  | Total | Boys ^b^ | Girls ^b^ |
| 1987 [1] | 7-15 | Taichung, Taiwan | School; Student/parent-reported | 37801 (78.0) | Ever: 420 (1.11); Current: 333 (0.88) | NA | NA |
| 1994 [1] | 7-15 | Taichung, Taiwan | School; Student/parent-reported | 75960 (83.0) | Ever: 1428 (1.88); Current:1155 (1.52) | NA | NA |
| 1994 [2] | 6-7 | Taipei, Taiwan | Elementary school; Parent-reported | 4806 (NA) | Ever: 1149 (23.9); Past year: 197 (4.1) | NA | NA |
| 1995 [3] | 13-14 | Hong Kong | Secondary school; Parent-reported | 4667 (97.0) | Ever: 707 (15.0); Past year: 210 (4.5) | Ever: 14.0; Past year: 4.3 | Ever: 17.0; Past year: 4.7 |
| 1995 [4] | 13-14 | Beijing | School; Student-reported | 4167 (99.0) | Ever: 429 (10.3); Past year: 958 (2.3) | NA | NA |
| 1995 [4] | 13-14 | Guangzhou | School; Student-reported | 3855 (99.6) | Ever:  705 (18.3); Past year: 50 (1.3) | NA | NA |
| 1995 [4] | 13-14 | Urumqi | School; Student-reported | 3207 (98.0) | Ever: 196 (6.1); Past year: 67 (2.1) | NA | NA |
| 1995 [4] | 13-14 | Shanghai | School; Student-reported | 3483 (99.0) | Ever: 240 (6.9); Past year: 42 (1.2) | NA | NA |
| 1995 [4] | 13-14 | Chongqing | School; Student-reported | 4296 (99.0) | Ever: 434 (10.1); Past year: 86 (2.0) | NA | NA |
| 1995 [5] | 6-7 | Hong Kong | Primary school; Parent-reported | 3618 (97.0) | Ever:  1017 (28.1); Past year: 152 (4.2) | Ever: 28.8; Past year: 3.6 | Ever: 27.2; Past year: 4.9 |
| 1996 [6] | 6-7 | Beijing | Primary school; Parent-reported | 4080 (99.2) | Ever: 155 (3.8); Past year:  114 (2.8) | Ever: 3.8; Past year: 2.7 | Ever: 3.7;  Past year: 2.9 |
| 1996 [6] | 6-7 | Urumqi | Primary school; Parent-reported | 3588 (98.6) | Ever: 126 (3.5);  Past year: 72 (2.0) | Ever: 3.8; Past year: 2.0 | Ever: 3.1; Past year: 2.0 |
| 1996 [7] | 12-14 | Taiwan^c^ | Middle school (nonsmokers); Parent-reported | 312873 (89.3) | Ever: 24632 (7.9) | 12628 (8.2) | 12004 (7.5) |
| 1996 [8] | 12-15 | Taiwan^c^ | Middle school; Parent-reported | 42919 (86.9) | Past year: 10472 (2.4) | 2.7 | 2.2 |
| 1998 [9] | 9-11 | Hong Kong | Primary school; Parent-reported | 3110 (97.0) | Past year:  118 (3.8)^d^ | NA | NA |
| 1998 [9] | 9-11 | Beijing | Primary school; Parent-reported | 4227 (92.0) | Past year:  93 (2.2)^d^ | NA | NA |
| 1998 [9] | 9-11 | Guangzhou | Primary school; Parent-reported | 3565 (93.0) | Past year:  64 (1.8)^d^ | NA | NA |
| 1998^e^ [10] | 3-6 | Taichung, Taiwan | Kindergarten; Parent-reported | 4373 (81.0) | Ever:  289 (6.6) | NA | NA |
| 2001 [8] | 12-15 | Taiwan^c^ | Middle school; Parent-reported | 10215 (87.0) | Past year:  412 (4.0) | 4.2 | 3.9 |
| 2001^e^ [11] | 10-18 | Taipei, Taiwan | Junior high school; Students/parent-reported | 8723 (NA) | Past year: Reported: 515 (5.9); Diagnosed: 340 (3.9) | NA | NA |
| 2001 [12] | 6-12 | Taiwan^c^ | Primary school; Parent-reported | 21291 (88.8) | Past year: 1195 (5.6) | 688 (6.1) | 507 (4.9) |
| 2001 [13] | 13-14 | Beijing | School; Student-reported | 3531 (99.0) | Ever: 434 (12.3); Past year: 64 (1.8) | NA | NA |
| 2001 [14] | 13-14 | Guangzhou | School; Student-reported | 3675 (96.0) | Ever: 620 (17.6) | NA | NA |
| 2001 [15] | 13-14 | Lhasa, Tibet | Junior high school; Student-reported | 3190 (100.0) | Ever: 45 (1.4); Past year: 12 (0.4) | Ever:25 (1.6); Past year: 8 (0.5) | Ever:20 (1.2); Past year: 2 (0.1) |
| 2002 [16] | 6-7 | Hong Kong | Primary school; Parent-reported | 4448 (95.0) | Ever:  1366 (30.7); Past year: 187 (4.2) | NA | NA |
| 2002 [2] | 6-7 | Taipei, Taiwan | Elementary school; Parent-reported | 4832 (NA) | Ever: 1271 (26.3); Past year: 411 (8.5) | NA | NA |
| 2002^e^ [17] | 7-12 | Kaohsiung, Taiwan | Primary school; Students and parent-reported | 1452 (90.86) | Past year:  103 (7.1) | NA | NA |
| 2002 [18] | 13-14 | Hong Kong | Secondary school; Parent/guardian-reported | 3321 (99.0) | Ever: 421 (13.0)  Past year:  120 (3.6) | NA | NA |
| 2002 [19] | 6-8 | Taoyuan, Taiwan | School; Students/parent-reported | 3079 (95.7) | Ever:  314 (10.2); Past year: 240 (7.8) | Ever: 165 (10.7); Past year: 124 (8.0) | Ever: 149 (9.7); Past year: 116 (7.6) |
| 2002 [19] | 13-15 | Taoyuan, Taiwan | School; Student-reported | 3111 (95.7) | Ever:  146 (4.7); Past year: 134 (4.3) | Ever:70(4.4); Past year:70 (4.4) | Ever:76(5.1); Past year:  64 (4.3) |
| 2002 [20] | 6-8 | Changhwa, Taiwan | Elementary school; Parent/guardian-reported | 7040 (89.4) | Ever:  1265 (18.0); Past year: 490 (7.0) | Ever:675 (18.6); Past year: 258 (7.1) | Ever:590 (17.3); Past year: 232 (6.8) |
| 2002 [1] | 7-15 | Taichung, Taiwan | School; Student/parent-reported | 11580 (81.0) | Ever: 388 (3.4); Current: 322 (2.8) | Current:171 (2.9); Ever: 206 (3.5) | Current:151 (2.6);  Ever: 182 (3.2) |
| 2003 [21] | 10-12 | Taichung, Taiwan | Elementary school; Parent/guardian-reported | 4197 (81.9) | Past year: 369 (8.8) | NA | NA |
| 2003 [21] | 13-15 | Taichung, Taiwan | Junior high school; Student-reported | 7677 (91.9) | Past year: 484 (6.3) | NA | NA |
| 2005^e^ [22] | 1-6 | Tianjin | Kindergartens and primary school; Parent/guardian-reported | 3708 (83.0) | Current:  108 (2.9) | NA | NA |
| 2005 [23] | 4-12 | Taiwan^c^ | Taiwan National Health Interview Survey (NHIS) | 2934 (100) | Ever:  497 (16.9) | NA | NA |
| 2005 [24] | 6-13 | Harbin | Elementary school; Parent/guardian-reported | 2900 (NA) | Past year:  136 (4.7) | NA | NA |
| 2005 [24] | 6-13 | Shanghai | Elementary school; Parent/guardian-reported | 4395 (NA) | Past year:  286 (6.5) | NA | NA |
| 2005 [24] | 6-13 | Guangzhou | Elementary school; Parent/guardian-reported | 3094 (NA) | Past year:  167 (5.4) | NA | NA |
| 2005 [24] | 6-13 | Xi'an | Elementary school; Parent/guardian-reported | 1653 (NA) | Past year:  73 (4.4) | NA | NA |
| 2005 [24] | 6-13 | Wuhan | Elementary school; Parent/guardian-reported | 2061 (NA) | Past year:  126 (6.1) | NA | NA |
| 2005 [24] | 6-13 | Chengdu | Elementary school; Parent/guardian-reported | 2848 (NA) | Past year:  122 (4.3) | NA | NA |
| 2005 [24] | 6-13 | Hohhot | Elementary school; Parent/guardian-reported | 2025 (NA) | Past year:  130 (6.4) | NA | NA |
| 2005 [24] | 6-13 | Urumqi | Elementary school; Parent/guardian-reported | 2033 (NA) | Past year:  120 (5.9) | NA | NA |
| 2006 [25] | 2-7 | Taiwan | Day care centers + kindergarten; Parent-reported | 14862 (68.0) | Ever:  2539 (17.6) | NA | NA |
| 2006 [26] | 0-14 | Hong Kong | Face to face interview; Parent-reported | 7393 (98.6) | Ever:  288 (11.4) | NA | NA |
| 2007 [27] | 6-8 | Taichung, Taiwan | School; Parent/legal guardian-reported | 4622 (79.1) | Ever:  1105 (23.9); Past year: 448 (9.7) | Ever: 590 (24.0) | Ever: 515 (22.5) |
| 2007 [2] | 6-7 | Taipei, Taiwan | Elementary school; Parent-reported | 24999 (94.6) | Ever:  7450 (29.8); Past year: 2675 (10.7) | Ever: 30.2; Past year: 11.1 | Ever: 29.3; Past year: 10.2 |
| 2007 [28] | 4-18 | Keelung, Taiwan | School + kindergarten; Parent-reported | 5351 (94.9) | Ever:  578 (10.8); Current eczema:  487 (9.1) | Ever: 11.7; Current eczema: 9.6 | Ever: 10.0; Current eczema: 8.5 |
| 2008 [29] | 5-11 | Beijing | School; Parent/guardian-reported | 4176 (92.3) | Ever:  871 (29.4);  Past year: 22 (1.1) | NA | NA |
| 2008 [30] | 3 | Taiwan^c^ | Home-interview; Mothers-reported | 19381 (80.1) | Ever:  2033 (10.5) | NA | NA |
| 2009 [31] | 0-14 | Beijing | School/kindergarten; Parent/guardian-reported | 10372 (98.6) | Ever:  2141 (20.6) | 1153 (21.1) | 988 (20.1) |
| 2009 [31] | 0-14 | Chongqing | School/kindergarten; Parent/guardian-reported | 9846 (97.2) | Ever:  1085 (10.0) | 596 (11.3) | 489 (10.7) |
| 2009 [31] | 0-14 | Guangzhou | School/kindergarten; Parent/guardian-reported | 4072 (90.9) | Ever:  294 (7.2) | 165 (7.5) | 129 (6.9) |
| 2011 [32] | 6-18 | Shijiazhuang | School; Parent/guardian-reported | 10338 (90.0) | Ever:  1220 (11.8); Past year: 155 (1.5) | Ever: 647 (12.7); Past year: 82 (1.6) | Ever: 573 (10.9); Past year: 73 (1.4) |
| 2012 [33] | 3-6 | Harbin | Kindergartens/daycare centers/primary school; Parent-reported | 2506 (64.1) | Ever:  829 (33.1);  Past year:  306 (12.2) | NA | NA |
| 2012 [33] | 3-6 | Urumqi | Kindergartens/daycare centers/primary school; Parent-reported | 4618 (81.7) | Ever:  707 (15.3);  Past year:  614 (13.3) | NA | NA |
| 2012 [33] | 3-6 | Beijing | Kindergartens/daycare centers/primary school; Parent-reported | 5876 (65.0) | Ever:  2039 (34.7);  Past year:  928 (15.8) | NA | NA |
| 2012 [33] | 3-6 | Shanghai | Kindergartens; Parent-reported | 15266 (85.3) | Ever:  3572 (23.4);  Past year: 2122 (13.9) | NA | NA |
| 2012 [33] | 3-6 | Nanjing | Kindergartens/daycare centers/primary school; Parent-reported | 4014 (65.7) | Ever:  1140 (28.4);  Past year:  429 (10.7) | NA | NA |
| 2012 [33] | 3-6 | Xi'an | Kindergartens/daycare centers/primary school; Parent-reported | 2020 (83.5) | Ever:  586 (29.0);  Past year:  166 (8.2) | NA | NA |
| 2012 [33] | 3-6 | Taiyuan | Kindergartens/daycare centers/primary school; Parent-reported | 3700 (82.2) | Ever:  503 (13.6);  Past year:  178 (4.8) | NA | NA |
| 2012 [33] | 3-6 | Wuhan | Kindergartens/daycare centers/primary school; Parent-reported | 2193 (91.4) | Ever:  570 (26.0);  Past year:  184 (8.4) | NA | NA |
| 2012 [33] | 3-6 | Changsha | Kindergartens/daycare centers/primary school; Parent-reported | 2727 (59.0) | Ever:  815 (29.9);  Past year:  265 (9.7) | NA | NA |
| 2012 [33] | 3-6 | Chongqing | Kindergartens/daycare centers/primary school; Parent-reported | 5299 (74.5) | Ever:  1611 (30.4);  Past year:  684 (12.9) | NA | NA |
| 2013^e^ [34] | 0-16 | Liuzhou^c^, Guangxi | Kindergartens/daycare centers/primary school; Parent-reported | 4856 (100.0) | Ever:  411 (8.5) | NA | NA |
| 2015^e^ [35] | 13-15 | Taiwan^c^ | Personal interview; Students/parent-reported | 74688 (NA) | Ever:  5676 (7.6) | NA | NA |
| 2015 [36] | 7-12 | Guangzhou | Face-to-face questionnaire; Students/parent-reported | 5542 (94.3) | Ever:  1890 (34.1) | NA | NA |
| 2015 [36] | 7-12 | Shaoguan, Guangdong | Face-to-face questionnaire; Students/parent-reported | 5139 (91.8) | Ever:  1331 (25.9) | NA | NA |

^a^ If the study was conducted between two years or among longer than two years, the least year were provided. ^b^ NA: not available. ^c^ The city where the study was conducted was not provided in the literature; we assumed the city where the authors’ institution was located. ^d^ Visible flexural dermatitis in last 12 months. ^e^ The year when the study was conducted was not provided in the literature; we assumed the year when the study was published.

**S2 Table**. Basic information for studies that were not based on the ISAAC questionnaire for infants, 1985-2015.

| Year ^a^ [Reference] | Age  (years) | City | Location and Inspected method | Sample size (response rate) | Prevalence, N (%) or % | | |
| --- | --- | --- | --- | --- | --- | --- | --- |
|  |  |  |  |  | Total | Boys^b^ | Girls^b^ |
| 2000 [37] | <1 | Yi-Meng, Shandong | Clinic survey + Hospitalization survey + Home survey; dermatologist examinations, Clinical Dermatology [38] and Practical Pediatric Dermatology [39] | 1566 (100.0) | Current: 418 (26.7) | NA | NA |
| 2003 [40] | 38.4 weeks | Taiwan^c^ | home-interview, mothers-reported; Taiwan National Birth Cohort Study | 1760 (86.0) | Ever:  118 (6.7) | NA | NA |
| 2003 [41] | <1 | Ru-Yang, Henan | Clinic survey + Hospitalization survey + Home survey; dermatologist examinations, Clinical Dermatology [38] | 1566 (100.0) | Current: 418 (26.7) | 1.9 | 1.4 |
| 2004 [42] | 0-3 | Taiyuan, Shanxi | parent/guardian-reported | 479 (100.0) | Ever:  169 (35.3) | 90 (39.5) | 79 (31.5) |
| 2006 [43] | 6-36 months | Taiwan^c^ | Taiwanese national registry data (Taiwan Birth Cohort Study, TBCS) | 16686 (69.0) | Ever:  1206 (7.2) | 725 (8.3) | 481 (6.1) |
| 2006 [44] | 6 months | Taiwan^c^ | Home-interview, mothers-reported | 20687 (NA) | Ever: 1438 (7.0) | 873 (8.1) | 565 (5.7) |
| 2007 [45] | 6-12 months | Tianjin | Da-Gang, parent/guardian-reported | 506 (100.0) | Ever:  383 (75.7) | NA | NA |
| 2008 [46] | 0-3 | Jiujiang, Jiangxi | Physical check-ups, parent/guardian-reported | 500 (100.0) | Ever: 249 (49.8) | NA | NA |
| 2012 [47] | 42 days | Beijing | Hospital, Physical examination [38] | 2982 (57.3) | Current:  1657 (57.3) | NA | NA |
| 2013 [48] | 0-1 | Chongqing | Hospital, parent-reported and examined by dermatologists [49] | 270 (100.0) | Current:  175 (64.8) | 97 (68.8) | 78 (60.5) |

^a^ If the study was conducted between two years or among longer than two years, the least year were provided.

^b^ NA: not available. ^c^ The city where the study was conducted was not provided in the literature; we assumed the city where the authors’ institution was located. ^d^ The year when the study was conducted was not provided in the literature; we assumed the year when the study was published.

**S3 Table**. Basic information for studies that were not based on the ISAAC questionnaire for preschool and school children, 1985-2015.

| Year ^a^ [Reference] | Age  (years) | City | Location and Inspected method | Sample size (response rate) | Prevalence, N (%) or % | | |
| --- | --- | --- | --- | --- | --- | --- | --- |
|  |  |  |  |  | Total | Boys^b^ | Girls^b^ |
| 1989 [50] | 3-10 | Hong Kong | kindergarten and primary school, parent-reported | 535 (89.0) | Ever: 37 (6.8) | NA | NA |
| 1992 [51] | 11-20 | Hong Kong | Secondary school, parent-reported and randomly skin test; standard respiratory questionnaires [52]. | 1062 (89.2) | Ever: 213 (20.1) | NA | NA |
| 1996 [53] | 0-14 | Zhengzhou, Henan | Hospital, Otolaryngology department; doctors-reported | 1710 (100.0) | Past year: 357 (20.9) | NA | NA |
| 1997 [54] | 6-21 | Hong Kong | primary and secondary school, student-reported and skin tests | 1006 (95.0) | Ever: 68 (6.8) | NA | NA |
| 2000 [55] | 0-5 | Taiwan^c^ | National Health Insurance Research Database (NHIRD) [56] | 10729 (100.0) | Ever:  719 (6.7) | NA | NA |
| 2004 [57] | 6-11 | Kaohsiung, Taiwan | school, clinical surveyed and examined by dermatologists | 4067 (52.0) | Current: 1.7 | NA | NA |
| 2005^d^ [58] | 9-20 | Shanxi^c^ | school, student-reported; Questionnaire [59] | 2116 (90.0) | Ever: 55 (2.6) | 37 (3.5) | 18 (1.8) |
| 2005 [60] | 6-11 | Penghu, Taiwan | School, clinical surveyed and examined by dermatologists | 3273 (71.9) | Current:  142 (4.3) | NA | NA |
| 2007 [61] | 3-17 | Pu-Tuo, Shanghai | School, student-reported | 5807 (46.5) | Ever: 48 (0.8) | NA | NA |
| 2008 [62] | 3-18 | Kunming, Yunnan | school, kindergarten, parent/guardian-reported [63] | 3675 (91.9) | Current:  9 (0.2) | 5 (0.3) | 4 (0.2) |
| 2010 [64] | 3-6 | Shanghai | kindergarten, parent-reported [63] | 10891 (95.8) | Ever:  904 (8.3) | 8.5 | 8.2 |
| 2011 [65] | 6-12 | Jiading, Shanghai | school, parent/guardian-reported [63] | 4784 (99.7) | Ever:  526 (10.9) | 291 (12.1) | 235 (9.9) |
| 2011 [66] | 0-6 | Dalian, Liaoning | kindergarten, Care doctor-reported [63] | 7872 (92.7) | Current: 212 (2.7) | 104 (2.8) | 108 (2.6) |

^a^ If the study was conducted between two years or among longer than two years, the least year were provided.

^b^ NA: not available. ^c^ The city where the study was conducted was not provided in the literature; we assumed the city where the authors’ institution was located. ^d^ The year when the study was conducted was not provided in the literature; we assumed the year when the study was published.

**S4 Table**. Basic information for studies which of eczema prevalence among common adult populations.

| Year ^a^ [Reference] | Age  (years) | City | Location and Inspected method | Sample size (response rate) | Prevalence, *N* (%) or % | | |
| --- | --- | --- | --- | --- | --- | --- | --- |
|  |  |  |  |  | Total | Male ^b^ | Female ^b^ |
| 1992 [67] | 12-20 | Kaiping, Guangdong | Secondary school, parent-reported, Questionnaire [39] | 737 (98.6) | Ever:  77 (10.4) | NA | NA |
| 1999 [68] | >65 | Taiwan^c^ | National Taiwan University Hospital (NTUH), computer records [43] | 16294 (100.0) | Ever:  9933 (58.7) | 5984 (62.6) | 3949 (53.7) |
| 2006 [69] | 17-45 | Tianjin | Tianjin University, student-reported | 3712 (57.0) | Past year:  310 (9.0) | NA | NA |
| 2006 [70] | 7-20 | Benxi, Liaoning | School; Student/parent-reported, questionnaire [71] | 4346 (86.9) | 52 (1.2) | 25 (1.34) | 27 (1.09) |
| 2007 [72] | 39.1±21.8 | Lishui, Zhejiang | Community-interview; participant-reported and examined by dermatologists [73] | 2023 (87.0) | 152 (7.5) | NA | NA |
| 2010 [74] | 1-87 | Hebei and Tianjin | face to face interview; participant-reported and examined by dermatologists [60] | 1524 (NA) | 92 (6.0) | 42 (5.5) | 50 (6.6) |
| 2008 [75] | mean age: 49.95 | Beijing | Community-interview; participant-reported and examined by dermatologists [73] | 2009 (85.7) | Current: 9.3 | 9.8 | 8.8 |
| 2010 [76] | 15-86 | Beijing | home-interview, participant-reported and examined by dermatologists [77] | 1443 (NA) | Current dermatitis: contact: 47 (3.3); seborrheic: 29 (2.0); atopic: 22 (1.5) | NA | NA |
| 2010 [76] | 15-86 | Shanghai | home-interview, participant-reported and examined by dermatologists [77] | 6036 (NA) | Current dermatitis: contact: 192 (3.2); seborrheic: 75 (1.2); atopic: 38 (0.6) | NA | NA |
| 2010 [76] | 15-86 | Guangzhou | home-interview, participants-reported and examined by dermatologists [77] | 1675 (NA) | Current dermatitis: contact: 85 (5.1); seborrheic: 60 (3.6); atopic: 35 (2.1) | NA | NA |
| 2014 [78] | Mean age: 51.8 | Beijing (Urban) | home-interview and examined by dermatologists[45] | 2786 (81.8) | Current:  279 (10.0) | NA | NA |
| 2014 [78] | Mean age: 44.2 | Beijing (urban border area) | home-interview and examined by dermatologists[45] | 3925 (79.7) | Current:  468 (11.9) | NA | NA |

^a^ If the study was conducted between two years or among longer than two years, the least year were provided.

^b^ NA: not available. ^c^ The city where the study was conducted was not provided in the literature; we assumed the city where the authors’ institution was located.

**S5 Table**. Studies of eczema prevalence in nurses and/or doctors in China.

| Year ^a^ [Reference] | Age ^b^  (years) | City | Location and Inspected method | Definition | Prevalence,  *N* (%) |
| --- | --- | --- | --- | --- | --- |
| 1996 ^c^ [79] | <20->40 | Hong Kong | Hospital; employees-reported | Atopic eczema, ever | 307 (20.9) |
| 2003 [80] | 33-38 | Shijiazhuang, Hebei | Hospital; nurses-reported;  questionnaire [81] | Hand dermatitis, in the past year | 50 (17.7) |
| 2005 ^c^ [82] | NA | Shijiazhuang, Hebei | Hospital; physicians-reported; questionnaire [81] | Hand dermatitis: symptoms (erythema, itching, vesiculation and scaling); ever | 47 (12.9) |
| 2007 ^c^ [83] | NA | Taichung,  Taiwan | Hospital; Medical workers-reported | latex glove-related contact dermatitis, ever | 435 (35.0) |
| 2008 [84] | 22-64 | Kaohsiung, Taiwan | Hospital; nurses-reported, Questionnaire [85] | Hand dermatitis, ever | 248 (21.9) |
| 2008 [86] | NA | Kaohsiung, Taiwan | Hospital; self-reported; ISAAC, United Kingdom Working Party (UKWP) [87-89] | Atopic dermatitis, past year | 90 (8.0) |
| 2009 [90] | NA | Hong Kong | Hospital; nurses-reported; the Nordic Occupational Skin Questionnaire (NOSQ-2002) [91] | Hand dermatitis, ever | 160 (22.1) |
| 2013 [92] | 28.6±7.2 | Jiangsu | Hospital; nurses-reported; questionnaire [81] | Hand dermatitis, in the past year | Reported:  95 (22.3); Diagnosed: 119 (27.9) |
| 2014 ^c^ [93] | NA | Harbin, Heilongjiang | Hospital; nurses-reported, NOSQ-2002 [91] | Hand dermatitis, ever | 183 (19.6) |

^a^ If the study was conducted between two years or among longer than two years, the least year were provided.

^b^ NA: not available. ^c^ The year when the study was conducted was not provided in the literature; we assumed the year when the study was published.

**S6 Table**. Studies of eczema prevalence in soldiers in China.

| Year^a^ [Reference] | Age^b^  (years) | City | Location and Inspected method | Definition | Prevalence,  *N* (%) |
| --- | --- | --- | --- | --- | --- |
| 1999 [94] | NA | Southeast coastal area^c^ | Special force corps; dermatologist examinations | Diagnosed dermatitis-eczema | 568 (17.97) |
| 2005 [95] | 17-22 | Tianjin^c^ | [Military](javascript:void(0);) [camp](javascript:void(0);)us for Armed Police Force A; Dermatologist examinations | Current diagnosed dermatitis-eczema; one year after training | 104 (9.49) |
| 2005 [95] | 17-22 | Tianjin^c^ | [Military](javascript:void(0);) [camp](javascript:void(0);)us for Armed Police Force B; Dermatologist examinations | Current diagnosed dermatitis-eczema | 2003: 96 (5.99)  2004: 65 (4.27) |
| 2006 [96] | NA | Tianjin | [Military](javascript:void(0);) hospital; Dermatology Clinic information; Dermatologist examinations | Diagnosed dermatitis-eczema | 982 (28.02) |
| 2008 [97] | 18-46 | Yingxiu,  Sichuan | Earthquake rescuing troops in Ying-Xiu; Dermatologist examinations | Diagnosed acute scrotum eczema and  Diagnosed acute eczema; two months after being to the Ying-Xiu earthquake stricken area | Acute scrotum eczema: 281 (25.02); and acute eczema: 179 (15.94) |
| 2009 [98] | 16-19 | Beijing | [Military](javascript:void(0);) [camp](javascript:void(0);)us; Dermatologist examinations dermatologist examinations | Current diagnosed dermatitis-eczema | 24 (5.81) |
| 2011 [99] | 18-39 | [Beijing-Tianjin-Hebei](javascript:void(0);) [region](javascript:void(0);)^c^ | Grass-root units of [military](javascript:void(0);); Dermatologist examinations | Current diagnosed dermatitis-eczema | 149 (15.9) |
| 2012 [99] | 17-38 | [Beijing-Tianjin-Hebei](javascript:void(0);) [region](javascript:void(0);)^c^ | Grass-root units of [military](javascript:void(0);); Dermatologist examinations | Current diagnosed dermatitis-eczema | 110 (15.1) |
| 2012^d^ [100] | 17-43 | Beijing^c^ | Pontoon bridge [military](javascript:void(0);) [camp](javascript:void(0);)us; Self-reported, Physical examination | Current diagnosed dermatitis-eczema | 112 (12.4) |

^a^ If the study was conducted between two years or among longer than two years, the least year were provided.

^b^ NA: not available. ^c^ The city where the study was conducted was not provided in the literature; we assumed the city where the authors’ institution was located. ^d^ The year when the study was conducted was not provided in the literature; we assumed the year when the study was published.

**S7 Table**. Studies of eczema prevalence in other special populations in China.

| Year^a^ [Reference] | Age  (years) | City^b^ | Location and Inspection method | Definition | Prevalence,  *N* (%) |
| --- | --- | --- | --- | --- | --- |
| 1991 [101] | >10 | Taiwan^c^ | Village near Nan-Sy town; fruit farmers/workers-reported | Diagnosed eczema, ever (hand dermatitis) | 37 (30.3) |
| 1994^d^ [102] | median age: 19 | Tainan, Taiwan | Hairdressing shop; on-site interviews, dermatologist examinations and patch test | Current diagnosed eczema (hand dermatitis) | 82 (83.7) |
| 1994 [103] | <69 | Tainan, Taiwan | Telephone-interview, cement workers-reported, skin examination | Current hand dermatitis | 57 (41.6) |
| 2000 [104] | 22-108 | Tainan, Taiwan | Nursing homes; patients' primary caregiver-report and skin examination | Personal history of dermatitis ever; at least one of the following symptoms were present: skin thickening, mild erythema, itching, hyperpigmentation, edema, vesicles, fissures, or marked erythema. | 29 (7.3) |
| 2004 ^d^ [105] | >10 | Taiwan^c^ | Semi-conductor manufacturing industry; electronics workers-reported | Symptoms (itching or redness/scaling) in the last 12 months | 302 (9.8) |
| 2008 [106] | 11-101 | Mianzhu + Mianyang + Deyang, Sichuan | Earthquake victims; Self-reported; dermatologist examinations | Diagnosed dermatitis eczema; 49 days after the earthquake | 1656 (15.25) |
| 2009 [107] | 32.9±10.7 | NA | Black metal smelting and rolling processing enterprises, Workers-reported; questionnaire [81] | Hand dermatitis, in the past year | 241 (12.8) |
| 2013 [108] | 14-75 | Fujian^c^ | Pig feeder; on-site interviews and dermatologist examinations | Current [contact](javascript:void(0);) [dermatitis](javascript:void(0);); Diagnostic Criteria for Occupational Contact Dermatitis [109] | 70 (23.2) |
| 2015 [110] | 20-55 | Gansu^c^ | Coal mine area; Workers-reported; dermatologist examinations | Current diagnosed dermatitis-eczema | 126 (18.3) |

^a^ If the study was conducted between two years or among longer than two years, the least year were provided.

^b^ NA: not available. ^c^ The city where the study was conducted was not provided in the literature; we assumed the city where the authors’ institution was located. ^d^ The year when the study was conducted was not provided in the literature; we assumed the year when the study was published.

**References**

[1] Liao PF, Sun HL, Lu KH, Lue KH. Prevalence of Childhood Allergic Diseases in Central Taiwan over the Past 15 Years. Pediatr Neonatol 2009; 50(1):18-25.

[2] Wu WF, Wan KS, Wang SJ, Yang W, Liu WL. Prevalence, Severity, and Time Trends of Allergic Conditions in 6-to-7-Year-Old Schoolchildren in Taipei. J Invest Allergy Clin 2011; 21(7):556-562.

[3] Leung R, Wong G, Lau J, Ho A, Chan JKW, Choy D. Prevalence of asthma and allergy in Hong Kong schoolchildren: an ISAAC study. Eur Respir J 1997; 10(2):354-360.

[4] Chen YZ, Zhao TB, Ding Y, Wang HJ, Wang HY, Zhong NS, et al. A questionnaire-based survey on prevalences of asthma, allergic rhinitis and eczema in five Chinese cities (ISAAC). Chin J Pediatr 1998; 36:352-355. (In Chinese)

[5] Lau YL, Karlberg J. Prevalence and risk factors of childhood asthma, rhinitis and eczema in Hong Kong. J Paediatr Child Health 1998; 34(1):47-52.

[6] Zhao TB, Wang HJ, Chen YZ, Xiao ML, Duo LK, Liu G. Prevalence of childhood asthma, allergic rhinitis and eczema in Urumqi and Beijing. J Paediatr Child Health 2000; 36(2):128-133.

[7] Lee YL, Shaw CK, Su HJ, Lai JS, Ko YC, Huang SL, et al. Climate, traffic-related air pollutants and allergic rhinitis prevalence in middle-school children in Taiwan. Eur Respir J 2003; 21(6):964-970.

[8] Lee YL, Li CW, Sung FC, Guo YL. Increasing prevalence of atopic eczema in Taiwanese adolescents from 1995 to 2001. Clin Exp Allergy 2007; 37(4):543-551.

[9] Wong GWK, Hui DSC, Chan HH, Fok TF, Leung R, Zhong NS, et al. Prevalence of respiratory and atopic disorders in Chinese schoolchildren. Clin Exp Allergy 2001; 31(8):1225-1231.

[10] Wang WC, Lue KH, Sheu JN. Allergic diseases in preschool children in Taichung City. Acta Paediatrica Sinica (Zhonghua Min Guo Xiao Er ke Yi Xue Hui Za Zhi) 1998; 39(5):314-318.

[11] Chen CF, Wu KG, Hsu MC, Tang RB. Prevalence and relationship between allergic diseases and infectious diseases. J Microbiol Immunol Infect 2001; 34(1):57-62.

[12] Lee YL, Li CW, Sung FC, Yu HS, Sheu HM, Guo YL. Environmental factors, parental atopy and atopic eczema in primary-school children: a cross-sectional study in Taiwan. Brit J Dermatol 2007; 157(6):1217-1224.

[13]Ma Y, Kang XH, Zhang JL. Prevalence of asthmatic and atopic disorders in Chinese schoolchildren in Beijing, a comparison between 2001 and 1994. Beijing Med J 2004; 26:112-115. (In Chinese)

[14]Wang HY, Zheng JP, Zhong NS. Time trends in the prevalence of asthma and allergic diseases over 7 years among adolescents in Guangzhou city. Zhonghua Yi Xue Za Zhi 2006; 86(15):1014-1020.

[15] Droma Y, Kunii O, Yangzom Y, Shan M, Pingzo L, Song P. Prevalence and severity of asthma and allergies in schoolchildren in Lhasa, Tibet. Clin Exp Allergy 2007; 37(9):1326-1333.

[16] Lee SL, Wong W, Lau YL. Increasing prevalence of allergic rhinitis but not asthma among children in Hong Kong from 1995 to 2001 (Phase 3 International Study of Asthma and Allergies in Childhood). Pediatr Allergy Immunol 2004; 15(1):72-78.

[17] Chen WY, Tseng HI, Wu MT, Hung HC, Wu HT, Chen HL. Synergistic effect of multiple indoor allergen sources on atopic symptoms in primary school children. Environ Res 2003; 93(1):1-8.

[18] Wong GWK, Leung TF, Ko FWS, Lee KKM, Lam P, Hui DSC. Declining asthma prevalence in Hong Kong Chinese schoolchildren. Clin Exp Allergy 2004; 34(10):1550-1555.

[19] Kao CC, Huang JL, Ou LS, See LC. The prevalence, severity and seasonal variations of asthma, rhinitis and eczema in Taiwanese schoolchildren. Pediatr Allergy Immunol 2005; 16(5):408-415.

[20] Liao MF, Huang JL, Chiang LC, Wang FY, Chen CY. Prevalence of asthma, rhinitis, and eczema from ISAAC survey of schoolchildren in central Taiwan. J Asthma 2005; 42(10):833-837.

[21] Chiang LC, Chen YH, Hsueh KC, Huang JL. Prevalence and severity of symptoms of asthma, allergic rhinitis, and eczema in 10-to 15-year-old schoolchildren in central Taiwan. Asian Pac J Allergy 2007; 25(1): 1-5.

[22] Zeng SW, Tang NJ, Ji LM, Coenraads PJ. Prevalence of atopic dermatitis in infants and children in Tianjin, China. J Invest Dermatol 2005; 125(4):854-854.

[23] Wu WC, Chang HY, Kuo KN, Chen CY, Tu YC, Yang YH. Psychosocial problems in children with allergic diseases: a population study in Taiwan. Child Care Health Dev 2011; 37(5):662-670.

[24] Li F, Zhou YC, Li SH, Jiang F, Jin XM, Yan CH, et al. Prevalence and risk factors of childhood allergic diseases in eight metropolitan cities in China: A multicenter study. BMC Public Health 2011; 11:437.

[25] Hsu NY, Wu PC, Bornehag CG, Sundell J, Su HJ. Feeding bottles usage and the prevalence of childhood allergy and asthma. Clin Dev Immunol 2012; 2012:158248. doi:10.1155/2012/158248.

[26] Lee SL, Lam TH, Leung TH, Wong WHS, Schooling M, Leung GM, et al. Foetal exposure to maternal passive smoking is associated with childhood asthma, allergic rhinitis, and eczema. Scientific World J 2012; 2012:542983.

[27] Liao MF, Liao MN, Lin SN, Chen JY, Huang JL. Prevalence of Allergic Diseases of Schoolchildren in Central Taiwan. J Asthma 2009; 46(6):541-545.

[28] Yao TC, Ou LS, Yeh KW, Lee WI, Chen LC, Huang JL, et al. Associations of Age, Gender, and BMI with Prevalence of Allergic Diseases in Children: PATCH Study. J Asthma 2011; 48(5): 503-510.

[29] Lv HB, Deng FR, Sun JD. The comparison of the indoor environmental factors associated with asthma and related allergies among school-child between urban and suburban area in Beijing. Chin J Prev Med. 2010; 7(44):626-630.

[30] Wang, IJ, Wen HJ, Chiang TL, Lin SJ, Chen PC, Guo YL. Maternal employment and atopic dermatitis in children: a prospective cohort study. Brit J Dermatol 2013; 168(4):794-801.

[31] Zhao J, Bai J, Shen KL, Xiang L, Huang S, Chen AH, et al. Self-reported prevalence of childhood allergic diseases in three cities of China: a multicenter study. BMC Public Health 2010; 10:551.

[32] Song N, Mohammed S, Zhang J, Wu JL, Fu CL, Hao ST, et al. Prevalence, severity and risk factors of asthma, rhinitis and eczema in a large group of Chinese schoolchildren. J Asthma 2014; 51(3):232-242.

[33] Zhang Y, Li BZ, Huang C, Yang X, Qian H, Deng QH, et al. Ten cities cross-sectional questionnaire survey of children asthma and other allergies in China. Chin Sci Bull 2013; 58(34):4182-4189.

[34] Wang J. Epidemiological survey on bronchial asthma, allergic rhinitis and eczema among urban children in Guangxi. Mat Child Health Care China 2014; 29(17):2779-2780. (In Chinese)

[35] Lin MH, Hsieh CJ, Caffrey JL, Lin YS, Wang IJ, Ho WC, et al. Fetal Growth, Obesity, and Atopic Disorders in Adolescence: a Retrospective Birth Cohort Study. Paediatr Perinat Epidemiol 2015; 29(5):472-479.

[36] Yang Z, Zheng W, Yung E, Zhong N, Wong GWK, Li J. Frequency of food group consumption and risk of allergic disease and sensitization in schoolchildren in urban and rural China. Clin Exp Allergy 2015; 45(12):1823-1832.

[37] Wang LY, Wang HJ, Meng J, Zhang YX. Investigation and analysis of infant eczema in Yi-Meng mountain area. Chin J Dermatol Venereol 2001; 15(6):382-383. (In Chinese)

[38] Zhao B. Clinical Dermatology, Second version. Jiangsu Science and Technology Publishing House, Nanjing, China. 1989; 512-520. (In Chinese)

[39] Tu YY, Yuan CY. Practical Pediatric Dermatology. Chongqing: Science and Technology Literature Press. 1986, 102-106. (In Chinese)

[40] Wang IJ, Guo YL, Weng HJ, Hsieh WS, Chuang YL, Lin SJ, et al. Environmental risk factors for early infantile atopic dermatitis. Pediatr Allergy Immunol 2007; 18(5):441-447.

[41] Zhang ZK. Investigation and analysis for the situation of infant eczema. J Med Forum 2006; 27(2): 42. (In Chinese)

[42] He YH, Kang J, Liu GZ, Chen GT. Analysis of factors relevant to the onset of eczema in 479 infants in Taiyuan. Chin J Info on TCM 2006. 13(8):12-13. (In Chinese)

[43] Huang CC, Wen HJ, Chen PC, Chiang TL, Lin SJ, Guo YL. Prenatal air pollutant exposure and occurrence of atopic dermatitis. Brit J Dermatol 2015; 173(4):981-988.

[44] Wen HJ, Chen PC, Chiang TL, Lin SJ, Chuang YL, Guo YL. Predicting risk for early infantile atopic dermatitis by hereditary and environmental factors. Brit J Dermatol 2009; 161(5):1166-1172.

[45] Liu J, Ye T, Li YM, Zhang LX, Xu BL. Epidemiological investigation on infant eczema symptoms. Mat Child Health Care China 2008; (21):3025-3026. (In Chinese)

[46] Hu HD. Analysis of related factors for infant eczema among 500 cases. Chin J Mod Drug Appl 2010; 4(4):105-106. (In Chinese)

[47] Shen CP, Yan Q, Wang ZY, Tian J, Ma XH, Zhang W, et al. Epidemiologic investigation and treatment for the younger infantile eczema. J Clin Dermatol 2015; 44(8):469-472. (In Chinese)

[48] Feng M, Xiao YZ, Luo XY, Hu Y. Investigations of environmental risk factors of eczema in different genetic background infants. Chin J Child Health Care 2015; 10:1070-1073. (In Chinese)

[49] Immune Group in the Skin Venereology Branch of Chinese Medical Association. Chinese guidelines for eczema diagnosis. Chin J Dermatol 2011; 44(1):5-6. (In Chinese)

[50] Lau YL, Karlberg J, Yeung CY. Prevalence of and factors associated with childhood asthma in Hong Kong. Acta Paediatr 1995; 84(7):820-822.

[51] Leung R, Ho P. Asthma, allergy, and atopy in three south-east Asian populations. Thorax 1994; 49(12):1205-1210.

[52] Samet JM. A historical and epidemiologic perspective on respiratory symptoms questionnaires. Am J Epidemiol 1978; 108: 435-436.

[53] Chen LL, Ma F, Han FG. Investigation and analysis of otolaryngology diseases among 4154 children in Zhengzhou. Henan J Pre Med 1997; 8(3):136-137. (In Chinese)

[54] Fung WK, Lo KK. Prevalence of skin disease among school children and adolescents in a Student Health Service Center in Hong Kong. Pediatr Dermatol 2000; 17(6):440-446.

[55] Sun HL, Yeh CJ, Ku MS, Lue KH. Coexistence of allergic diseases: patterns and frequencies. Allergy Asthma Proc 2012; 33(1): e1-e4.

[56] St Anthony’s ICD-9-CM Code Book. VA. St. Anthony Publishing. 1991.

[57] Yang YC, Cheng YW, Lai CS, Chen W. Prevalence of childhood acne, ephelides, warts, atopic dermatitis, psoriasis, alopecia areata and keloid in Kaohsiung County, Taiwan: a community-based clinical survey. J Eur Acad Dermatol 2007; 21(5):643-649.

[58] Norbäck D, Zhao ZH, Wang ZH, Wieslander G, Mi YH, Zhang Z. Asthma, eczema, and reports on pollen and cat allergy among pupils in Shanxi province, China. Int Arch Occup Environ Health 2007; 80(3): 207-216.

[59] Smedje G, Norbäck D, Edling C. Asthma among secondary school pupils in relation to the school environment. Clin Exp Allergy 1997; 27:1270–1278.

[60] Chen GY, Cheng YW, Wang CY, Hsu TJ, Hsu MML, Yang PT, et al. Prevalence of skin diseases among schoolchildren in Magong, Penghu, Taiwan: A community-based clinical survey. J Formos Med Assoc 2008; 107(1):21-29.

[61] Shi YM, Zhang FY, Dai HL, Zhang SZ, Hang JQ. Prevalence of atopic disorders in school children in Changzheng town of Putuo district, Shanghai. Chin J General Practice 2010; 11:1436-1438. (In Chinese)

[62] Wang MH, Deng DQ, Fan YJ, Xie H, Xu YM, Han YT. Investigation on childhood atopic dermatitis in Kunming. Chin J Public Health 2011; 11:1468. (In Chinese)

[63] Williams HC, Burney PG, Pembroke AC, Hay RJ. Validation of the UK diagnostic criteria of atopic dermatitis in a population setting. U.K. Diagnostic Criteria for Atopic Dermatitis Working Party. Br J Dermatol 1996; 135:12-17.

[64] Xu F, Yan SX, Li F, Cai MQ, Chai WH, Wu MM, et al. Prevalence of Childhood Atopic Dermatitis: An Urban and Rural Community-Based Study in Shanghai, China. PLoS One 2012; 7(5):e36174.

[65] Xu F, Yang C, Chai WH, Peng WQ, Yan SX, Xu JH, et al. Childhood atopic dermatitis and Household Environmental risk factors: a cross-sectional study in 4784 children in Jiading district, Shanghai. J Environ Health 2012; 29(06):517-520. (In Chinese)

[66] Wei FL, Shi XM, Huang Y, Li EN, Yan MJ, Ren J. Epidemiological survey of atopic dermatitis among pre-school children in Dalian. Chin J Leprosy Skin Dis 2012; 28(11):779-782.

[67] Leung R, Jenkins M. Asthma, allergy and atopy in southern Chinese school students. Clin Exp Allergy 1994; 24(4):353-358.

[68] Liao YH, Chen KH, Tseng MP, Sun CC. Pattern of skin diseases in a geriatric patient group in Taiwan: A 7-year survey from the outpatient clinic of a University Medical Center. Dermatology 2001; 203(4):308-313.

[69] Sun Y, Zhang Y, Sundell J, Fan Z, Bao L. Dampness at dorm and its associations with allergy and airways infection among college students in China: a cross-sectional study. Indoor Air 2009; 19(2): 174-182.

[70] Zhao LP, Jiang H, Zhang L, Hong YX, Song Y, Chen HD, et al. Epidemiological survey of atopic dermatitis in youngsters in Benxi. Chin J Dermatol Venereol 2013; 27(6):601-604. (In Chinese)

[71] Popescu CM, Popescu R, Williams H, Forsea D. Community validation of the United Kingdom diagnostic criteria for atopic dermatitis in Romanian schoolchildren. Br J Dermatol 1998; 138(3):436-442.

[72] Lu XY, Li LF, You YM. Prevalence of and risk factors for skin diseases in a community of Lishui City. China J Lepr Skin Dis 2008; 24(9): 692-694. (In Chinese)

[73] Li LF. Contact Dermatitis and Skin Allergy, Second version. Joint publishing house of Beijing Medical University and China Xie-He Medical University. 2003.02 (In Chinese)

[74] Wang ZH, Lin WS, Li SY, Zhao SC, Wang L, Yang ZG. Analysis of the correlation of prevalence in allergic rhinitis and other allergic diseases. Chin J Otorhinolaryngol Head Neck Surg 2012; 47(5):379-382. (In Chinese)

[75] You YM, Li LF. The prevalence of skin diseases in a community of Beijing and analysis of risk factors. Chin J Dermatol Venereol 2011; 25(6): 459-461. (In Chinese)

[76] Xu F, Yan S, Wu M, Li F, Sun Q, Lai W, et al. Self-declared sensitive skin in China: a community-based study in three top metropolises. J Eur Acad of Dermatol 2013; 27(3):370-375.

[77] Willis CM, Shaw S, De Lacharrière O, Baverel M, Reiche L, Jourdain R, et al. Sensitive skin: an epidemiological study. Br J Dermatol 2001; 145:258-263.

[78] Li J, You YM, Feng H. Prevalence of chronic eczema in urban and urban fringe of one district in Beijing and analysis of risk factors. China J Modern Med 2015; 25(20):73-77. (In Chinese)

[79] Leung R, Ho A., Chan J., Choy D, Lai CKW. Prevalence of latex allergy in hospital staff in Hong Kong. Clin Exp Allergy 1997; 27(2):167-174.

[80] Smith DR, Wei N, Zhao L, Wang RS. Hand dermatitis among nurses in a newly developing region of Mainland China. Int J Nurs Study 2005; 42(1):13-19.

[81] Smit HA, Coenraads PJ, Lavrijsen APM, Nater JP. Evaluation of a self-administered questionnaire on hand dermatitis. Contact Dermatitis 1992; 26:11-16.

[82] Smith DR, Wei N, Zhang YJ, Wang RS. Hand dermatitis among a complete cross-section of Chinese physicians. Contact Dermatitis 2005; 52(5):291-293.

[83] Lin CT, Hung DZ, Chen DY, Wu HJ, Lan JL, Chen YH. A hospital-based screening study of latex allergy and latex sensitization among medical workers in Taiwan. J Microbiol Immunol 2008; 41(6):499-506.

[84] Lan CCE, Feng WW, Lu YW, Wu CS, Hung ST, Hsu HY, et al. Hand eczema among University Hospital nursing staff: identification of high-risk sector and impact on quality of life. Contact Dermatitis 2008; 59(5): 301-306.

[85] Flower J, Ghosh A, Sung J, Emani S, Chang J, Den E, et al. Impact of chronic hand dermatitis on quality of life, work productivity, activity impairment, and medical costs. J Am Acad Dermatol 2006; 54:448–487.

[86] Lan CCE, Lee CH, Lu YW, Lin CL, Chiu HH, Chou TC, et al. Prevalence of adult atopic dermatitis among nursing staff in a Taiwanese medical center: a pilot study on validation of diagnostic questionnaires. J Am Acad Dermatol 2009; 61(5):806-812.

[87] Williams HC, Burney PG, Hay RJ, Archer CB, Shipley MJ, Hunter JJ, et al. The U.K. Working Party’s Diagnostic Criteria for Atopic Dermatitis. I: Derivation of a minimum set of discriminators for atopic dermatitis. Br J Dermatol 1994; 131:383-396.

[88] Williams HC, Burney PG, Strachan D, Hay RJ. The U.K. Working Party’s Diagnostic Criteria for Atopic Dermatitis. II: Observer variation of clinical diagnosis and signs of atopic dermatitis. Br J Dermatol 1994; 131:397-405.

[89] Williams HC, Burney PG, Pembroke AC, Hay RJ. The U.K. Working Party’s Diagnostic Criteria for Atopic Dermatitis. III: Independent hospital validation. Br J Dermatol 1994; 131:406-16.

[90] Luk NMT, Lee HCS, Luk CKD, Cheung YYA, Chang MC, Chao VKD, et al. Hand eczema among Hong Kong nurses: a self-report questionnaire survey conducted in a regional hospital. Contact Dermatitis 2011; 65(6):329-335.

[91] Susitaival P, Flyvholm MA, Meding B, Kanerva L, Lindberg M, Svensson A, et al. Nordic Occupational Skin Questionnaire (NOSQ-2002): a new tool for surveying occupational skin diseases and exposure. Contact Dermatitis 2003; 49:70–76.

[92] Liu LP, Li YM, Xu H, Ma H. Self-administered questionnaire on hand eczema in nurses. J Clin Dermatol 2014; 43(5):282-285. (In Chinese)

[93] Zhang D. Correlation analysis on the pathogenic factors for nurse hand eczema. Latest med info Essay (Electronic edition) 2014; 14(22): 179-180. (In Chinese)

[94] Che DF, Ni RZ, Cao Y, Dai FC, Liang XB, Ni T, et al. Investigation and prophylaxis of the high-morbidity dermatoses in a certain special army unit in the southeast of China. J Med Postgraduates 2001; 14(5): 382-384. (In Chinese)

[95] Niu YT, Yang XQ, Dai EQ, Lin CL, Xu HH, Bai Y, et al. Relationship between military training and dermatitis in armed police forces soldiers. Med J Chin People’s Armed Police Forces 2007; 18(6): 414-416. (In Chinese)

[96] Lu T, Niu YT, Xu HH, Lin CL, Wang W. An investigation and analysis of 3748 military medical fee-free out-patients in a troops hospital dermatological department. Acta Acad Med CPAF 2011; 20(9): 692-693+713. (In Chinese)

[97] Shi YX, Xu H, Qiu J, Yan LS, Zhang YF, Tang DH, et al. Analyze traumas and diseases in part of the soldiers of the earthquake-rescue troops in Yingxiu town and our experience on treatment and prevention. J Navy Med 2009; 30(4): 333-336. (In Chinese)

[98] Chen WH, Li XP, Gu XF, Liu J, Xu DH. Investigation on skin disease among Navy flying cadets before and after training. Military Med J Southeast China 2011; 13(5):421-424. (In Chinese)

[99] Tian YL, Wang WL, Song KM, Su YM, Peng SW. Analysis on the incidence and distribution of dermatoses among officers and soldiers in area of Beijing, Tianjin and Hebei Province. J Pract Dermatol 2012; 5(4):208-209. (In Chinese)

[100] Zhu H, Yang RY. Dermatopathic investigation of northern China pontoon bridge troop in the summer. J Pract Dermatol 2013; 6(4):210-211. (In Chinese)

[101] Guo YL, Wang BJ, Lee CC, Wang JD. Prevalence of dermatoses and skin sensitisation associated with use of pesticides in fruit farmers of southern Taiwan. Occup Environ Med 1996; 53(6):427-431.

[102] Guo YL, Wang BJ, Lee JYY, Chou SY. Occupational hand dermatoses of hairdressers in Tainan City. Occup Environ Med 1994; 51(10): 689-692.

[103] Guo YL, Wang BJ, Yeh KC, Wang JC, Kao HH, Wang MT, et al. Dermatoses in cement workers in southern Taiwan. Contact Dermatitis 1999; 40(1):1-7.

[104] Smith DR, Sheu HM, Hsieh FS, Lee YL, Chang SJ, Guo YL, et al. Prevalence of skin disease among nursing home patients in southern Taiwan. Int J Dermatol 2002; 41(11):754-759.

[105] Shiao JSC, Sheu HM, Chen CJ, Tsai PJ, Guo YL. Prevalence and risk factors of occupational hand dermatoses in electronics workers. Toxicol Ind Health 2004; 20:1-7.

[106] Nie YL, Jiang SS, Zhang R, Wang KP, Liu N, Wang R, et al. Spectrum of diseases in 15 towns of earthquake attacked areas in Sichuan province. Med J Chin PLA 2009; 34(2):232-234. (In Chinese)

[107] Jia N, Qin RL, Li YZ, Zhang XY, Li JG, Zhao CX, et al. Hand eczema and its risk factors of workers in ferrous metal smelting and Calendering processing industry. J Environ Occup Med 2012; 29(7):415-419. (In Chinese)

[108] Zhang YF, Lu ZY, Xie D, Gu Y, Chen MX, Li XY, et al. Analysis of risk factors for contact dermatitis in pig farm workers. Chin J Ind Hyg Occup Dis 2014; 32(2):120-122. (In Chinese)

[109] GBZ 20-2002. Diagnostic Criteria of Occupational Contact Dermatitis.

[110] Zhang JX, He YY, Liu HZ. Investigation on the risk factors of eczema in coal mine workers. Chin Community Doctors 2015; 23:75-75, 77.
